# Durable superhydrophobic surface in wearable sensors: From nature to application

**DOI:** 10.1002/EXP.20230046

**Published:** 2023-11-08

**Authors:** Ziyi Dai, Ming Lei, Sen Ding, Qian Zhou, Bing Ji, Mingrui Wang, Bingpu Zhou

**Affiliations:** ^1^ Joint Key Laboratory of the Ministry of Education Institute of Applied Physics and Materials Engineering University of Macau Avenida da Universidade Taipa Macau China; ^2^ State Key Laboratory of Crystal Materials Institute of Novel Semiconductors School of Microelectronics Shandong University Jinan China; ^3^ School of Physics and Electronics Central South University Changsha China; ^4^ School of Physics and Electronics Hunan Normal University Changsha China; ^5^ Department of Mechanical Engineering University of Auckland Auckland New Zealand

**Keywords:** encapsulation‐free devices, superhydrophobic surfaces, wearable sensors

## Abstract

The current generation of wearable sensors often experiences signal interference and external corrosion, leading to device degradation and failure. To address these challenges, the biomimetic superhydrophobic approach has been developed, which offers self‐cleaning, low adhesion, corrosion resistance, anti‐interference, and other properties. Such surfaces possess hierarchical nanostructures and low surface energy, resulting in a smaller contact area with the skin or external environment. Liquid droplets can even become suspended outside the flexible electronics, reducing the risk of pollution and signal interference, which contributes to the long‐term stability of the device in complex environments. Additionally, the coupling of superhydrophobic surfaces and flexible electronics can potentially enhance the device performance due to their large specific surface area and low surface energy. However, the fragility of layered textures in various scenarios and the lack of standardized evaluation and testing methods limit the industrial production of superhydrophobic wearable sensors. This review provides an overview of recent research on superhydrophobic flexible wearable sensors, including the fabrication methodology, evaluation, and specific application targets. The processing, performance, and characteristics of superhydrophobic surfaces are discussed, as well as the working mechanisms and potential challenges of superhydrophobic flexible electronics. Moreover, evaluation strategies for application‐oriented superhydrophobic surfaces are presented.

## Graphical abstract

This review provides a thorough overview of the latest advancements in the field of superhydrophobic flexible wearable sensors. The review encompasses a wide range of aspects including the manufacturing processes, evaluation methods, and specific applications of flexible sensors that have been functionalized with non‐wetting behavior. Furthermore, the review summarizes the various working mechanisms of superhydrophobic and flexible devices, followed with a detailed discussion of the potential challenges that may arise during the real‐world implementation.

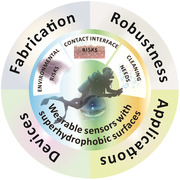

## 1 INTRODUCTION

Functional electronics have reached a state‐of‐the‐art level, paving the way for the development of metaverse technology. This technology is a reflection of the public's desire for an upgraded immersive and interactive experience, allowing them to break free from physical limitations and enter a parallel electrical twin environment since 2021.^[^ ^1^, ^2^, ^3^ ^]^ The booming embodied virtuality (EV) industry has also brought attention to flexible electronics and artificial intelligence.^[^ ^4^, ^5^ ^]^ With computer equipment expanding from desktop PCs to pervasive smart terminals that can be integrated into physical environments, traditional human–machine interfaces like mouse and keyboards are being replaced by wearable electronics that provides controllability, mobility, and convenience. The revolution in communication between humans and machines is a response to the demand for a trendy, intelligent, and data‐driven lifestyle, which spans across various fields such as healthcare,^[^ ^6^ ^]^ medical monitoring,^[^ ^7^ ^]^ entertainment,^[^ ^8^ ^]^ and the military.^[^ ^9^ ^]^ Wearable sensors are a new class of devices that can overcome environmental constraints and embed applications into the landscape of daily living. As such, they represent a double level of meanings as interfaces. Firstly, they function as human–computer interfaces by converting environmental inputs, such as signals from analytical chemistry and applied physics,^[^ ^10^ ^]^ into various electrical impulses based on their inherent principles such as piezoresistive,^[^ ^11^ ^]^ capacitive,^[^ ^12^ ^]^ piezoelectric,^[^ ^13^ ^]^ and chemical.^[^ ^14^ ^]^ Secondly, wearable sensors are characterized by thin films that are always placed between the surface of the human body and the external environment, as well as the in vivo environment inside the body, serving as a physical interface for stimuli monitoring and signal transformation. Sensing interfaces located inside or outside the body are exposed to varying environmental conditions on either the side facing the skin or the side facing the environment. Consequently, minimizing the interference becomes a primary concern in such scenarios.^[^ ^15^, ^16^ ^]^ The internal surface of sensing interfaces, that is, the side in contact with the skin, is exposed to various secretions of the human body, such as moisture, corrosive acids, alkalis, salts, biomass, exfoliated cutin, and dust, which may continuously accumulate on the sensor surface after long‐term use. Similarly, the external environment, especially under extreme conditions such as underwater or high‐humidity environments, may result in unpredictable temporary or permanent functional degradation of flexible electronics due to pollution, corrosion, and mechanism failure.^[^ ^17^, ^18^ ^]^ Deliberate washing or cleaning also threatens the stability of wearable sensors. In order to ensure reliable and precise signal acquisition, wearable sensors are expected to possess essential features such as anti‐interference and self‐cleaning capabilities. Moreover, to facilitate their applicability in diverse scenarios, extreme conditions, such as underwater,^[^ ^19^ ^]^ low temperature, high temperature,^[^ ^20^ ^]^ etc., should also be considered as working circumstances for the soft interface to be used for broad applicability. Therefore, new interface technologies for lossless signals are urgently needed to ensure the stability of wearable sensors in various complex environments and time dimensions.

To mitigate potential risks in the development of intelligent human‐computer interaction interfaces, non‐wetting and self‐cleaning superhydrophobic surfaces have been proposed as a promising strategy to enable stable and widespread signals for advanced artificial intelligence systems. It has been reported that superhydrophobic surfaces possess remarkable properties, including self‐cleaning,^[^ ^21^ ^]^ water repellency,^[^ ^22^ ^]^ anti‐fog,^[^ ^23^ ^]^ anti‐icing,^[^ ^24^ ^]^ antibacterial,^[^ ^25^ ^]^ and anti‐corrosion.^[^ ^26^ ^]^ These properties make superhydrophobic surfaces an attractive option for minimizing interference and maintaining signal stability. For example, the anti‐adhesion capability of sensor interface can be positive to reduce lethal embolism and life‐threatening if the sensor is implanted for in vivo applications. Milionis et al. have identified that a superhemophobic surface with low adhesion to complex droplets (e.g., proteins, glucose, mineral ions, hormones, carbon dioxide, oxygen, and suspended blood cells) could be a promising candidate for the development of a diagnostic platform and auxiliary material for prosthetic implants. Sensors, located on the skin surface, require stable working performance under the action of various endocrine substances, including transparency, conductivity, and micro‐nano structure.^[^ ^27^ ^]^ Ding et al. have reported that the long‐term corrosion of the sensor's main structural elements by sweat caused by inorganic salt ions is not negligible. Wearable sensors with superhydrophobicity can significantly reduce the risk of short circuits when working in conductive solutions.^[^ ^28^ ^]^ Ni and colleagues also reported the significance of corrosion resistance of wearable sensor surfaces in deep‐sea environments, because it can significantly increase the corrosion resistance of equipment used for underwater operations.^[^ ^29^ ^]^

This strategy is inspired by the fascinating bioinspired phenomenon known as the lotus effect, named after the behavior of liquid droplets that effortlessly roll on the surface of a lotus leaf.^[^ ^30^ ^]^ Following the systematic exploration of superhydrophobicity in natural systems, a plethora of methods have emerged to fabricate synthetic superhydrophobic materials, encompassing both bottom‐up^[^ ^31^ ^]^ and top‐down^[^ ^32^ ^]^ fabrication approaches. Leveraging these technological breakthroughs, there have been numerous reports of functional devices. In 2022, Wang et al. provided a comprehensive review that focuses on sensor devices equipped with surface‐wetting functionalities at the skin interface. The review underscored the performance metrics and merits of devices that incorporated surfaces with diverse wetting properties in recent years.^[^ ^33^ ^]^ However, despite the success in obtaining superhydrophobicity through various fabrication methods, the practical implementation of such materials in industrial applications remains difficult. Considering the fusion with wearable sensors to obtain working stability in complex environments, the application‐oriented superhydrophobic interface technology remains challenging, while provides a significant solution to ensure the reliable function of wearable device in the future. Hence, guided by these principles, application‐oriented design strategies should be embraced and supplemented by appropriate robustness testing methodologies to ascertain the potential for industrial viability and practical applications.

Superhydrophobicity relies on micro‐ or even nano‐scale surface topography with low surface energy, resulting in trapped air layers upon contact with water, that is, only a small portion of the entire area is in contact with water. Besides the high cost and complexity of process and manufacture, the hierarchical structure of superhydrophobic surfaces is also susceptible to defects caused by even small mechanical loads due to high local pressure on the surface texture.^[^ ^34^ ^]^ This, in turn, may lead to the elimination of superhydrophobicity, where water droplets are no longer repelled, but stick to defect area with high adhesion. The fragility of superhydrophobic technology poses a significant challenge for its real‐life applications. Specifically, the non‐wetting and self‐cleaning properties of superhydrophobic materials need to remain stable after geometric expansion due to the flexibility of the substrate.^[^ ^35^ ^]^ The preserved behavior is especially important for wearable sensing applications because such devices are always subjected to morphological deformations such as compression, stretching, and torsion. Furthermore, the application of superhydrophobic protective layers as insulating barriers and self‐cleaning mechanisms in wearable sensors is necessary to address the challenges posed by complex environment and diverse interactive manipulation like touching, bending, and swiping. At the same time, in order to wearable devices characterized by thin films, the roughness and low surface energy required for superhydrophobic surfaces are often designed in combination.^[^ ^36^ ^]^ In other words, materials and structures that form roughness and low surface energy often also serve as active components for sensors. Hence, it is crucial to develop a reliable and durable solution to ensure prolonged stability of the superhydrophobic interface in flexible electronics against mechanical wear and chemical corrosion, as well as to maintain sensing performance. Currently, numerous robust superhydrophobic surfaces have been developed based on different mechanisms of the wetting transition (WT) from the Cassie–Baxter to the Wenzel state.^[^ ^37^, ^38^ ^]^ A common approach is to use inherently abrasion‐resistant and stable hydrophobic materials to mitigate damage to surface texture and chemical composition.^[^ ^39^, ^40^ ^]^ For flexible substrates, the elastic component can also distribute localized stresses. Additionally, some superhydrophobic surfaces with self‐healing properties have been proposed to actively address failures induced by external corrosion.^[^ ^26^, ^41^ ^]^ The second key aspect concerns the integration of superhydrophobicity with the performance of wearable sensors. Specifically, when constructing superhydrophobic surfaces for use in wearable sensors, it is essential to ensure that the resulting surface does not compromise the sensing performance, including flexibility, stretchability,^[^ ^42^ ^]^ sensitivity,^[^ ^43^ ^]^ response time,^[^ ^44^ ^]^ long‐term stability,^[^ ^36^ ^]^ resolution,^[^ ^45^ ^]^ repeatability,^[^ ^46^ ^]^ and information capacity.^[^ ^47^ ^]^ These properties are also protected in some extreme environments like underwater by the “silver armor” of the Cassie–Baxter state, also known as the silver mirror effect, attributed to the refraction of the air–water film in the underwater non‐wetting state.^[^ ^48^ ^]^ However, it is important to also consider failure mechanisms when superhydrophobic surfaces are applied in extreme environments.^[^ ^49^ ^]^ For instance, underwater environments are subject to increasing water pressure with depth, which can result in rupture of the air‐pockets within the hierarchical structures of the superhydrophobic surface.^[^ ^50^ ^]^ Additionally, drops with low‐temperature can lead to the formation of condensation droplets, which may also cause wetting.^[^ ^51^ ^]^ Similar challenges arise in high humidity environments.^[^ ^52^ ^]^ Therefore, it is necessary to develop superhydrophobic surfaces that can maintain their performance in such extreme environments, which makes wearable sensors attractive for long‐term use in extreme environments.^[^ ^53^, ^54^ ^]^ Moreover, there is a growing interest in incorporating additional functions into superhydrophobic surfaces to enhance their overall functionality.^[^ ^55^ ^]^ The design principle for coupling superhydrophobic surfaces with wearable electronic devices relies on understanding the mechanisms of action and failure of the sensors. For example, there have been in‐depth works on the coupling effects in strain sensors,^[^ ^56^ ^]^ chemical sensors,^[^ ^57^ ^]^ and sensors based on triboelectronic nanogenerators.^[^ ^58^ ^]^ In addition to self‐cleaning and waterproofing, superhydrophobic surfaces are often related to properties including oil‐water separation, anti‐icing, anti‐fogging, and the larger surface area brought about by surface roughness.

**FIGURE 1:**
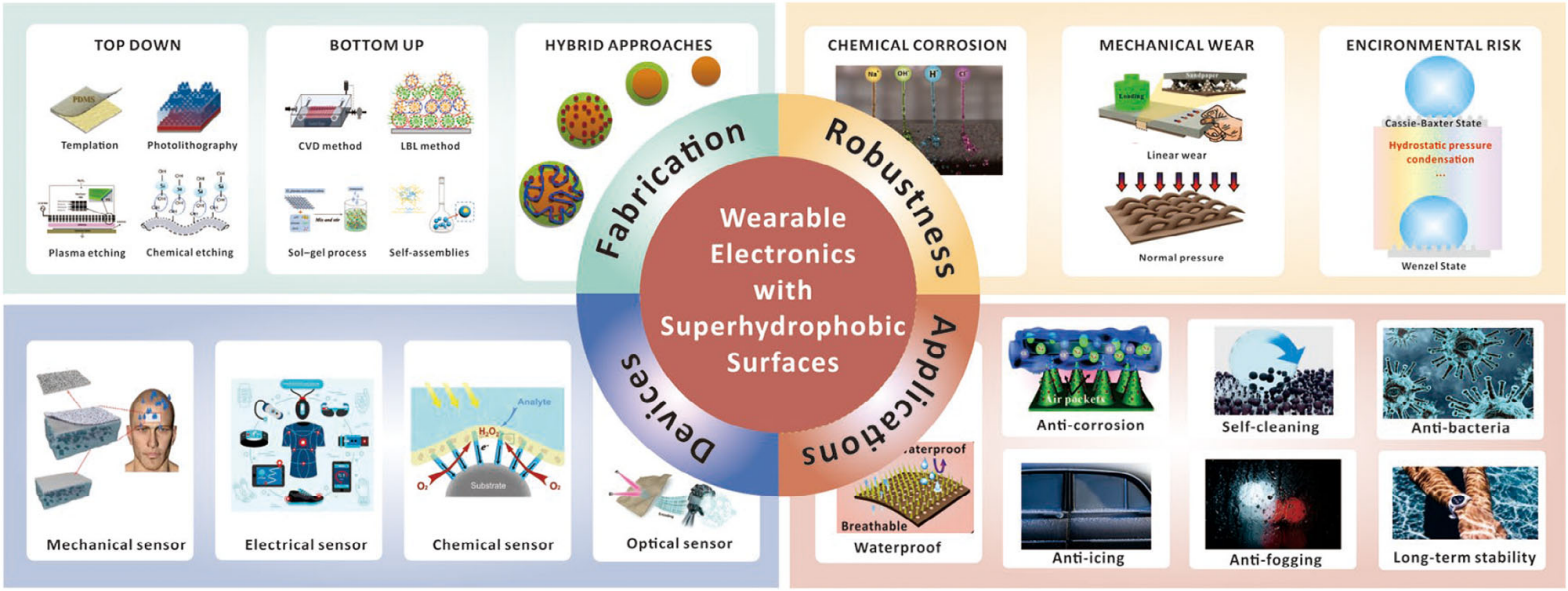
The schematic of wearable sensors that are coupled with a superhydrophobic surface. The figure highlights the preparation and device classification that showcase the fusion processing strategy of superhydrophobic flexible wearable sensors with diverse mechanisms. It also emphasizes the practical application of robust superhydrophobic surfaces for wearable sensing.

The investigation of superhydrophobicity in flexible electronics has become a state‐of‐art research area, as evidenced by the increasing number of publications in recent years.^[^ ^44^, ^59^, ^60^, ^61^ ^]^ Through reviewing related studies, it has been observed that the application of superhydrophobic surfaces inspired by nature in wearable electronics has sparked the interest of scientists from diverse fields, such as materials science, chemistry, electronics, and artificial intelligence. Through the effective control of solid or liquid interference on the surface, this development provides a solid foundation for the further advancement of universally applicable, distributed multi‐scenario sensing units in the metaverse, and their implementation in various domains, including healthcare, military affairs, fitness, entertainment, and emergency response.^[^ ^62^, ^63^ ^]^ As shown in Figure 1, this review aims to present a comprehensive overview of the latest development of durable superhydrophobic surfaces in conjunction with flexible electronics, as well as the assessment methods and strategies for their robustness, and future prospects for superhydrophobic wearable sensors. In this review, we will first provide an overview of fundamental aspects related to surface wetting properties, including theoretical analysis and fabrication methods. Next, we conduct a comprehensive study and review of the coupling development between flexible electronics and superhydrophobic interfaces with a detailed classification. Additionally, we emphasize two important aspects of superhydrophobic flexible electronics that are crucial for practical applications, namely, robustness and failure mechanisms in extreme environments. Finally, we offer our own perspective on the current status and future challenges in this field. Given the multidisciplinary nature of the topic, this review can serve as a starting point for researchers in fields such as materials science, chemistry, physics, electronics, and others, who are interested in designing and constructing robust superhydrophobic wearable sensing systems. We expect that this review can contribute to the advancement of flexible wearable superhydrophobic devices suitable for complex environments, leading to their integration into our daily lives.

## 2 SUPERHYDROPHOBICITY

### 2.1 Wetting theories of superhydrophobic surfaces

**FIGURE 2:**
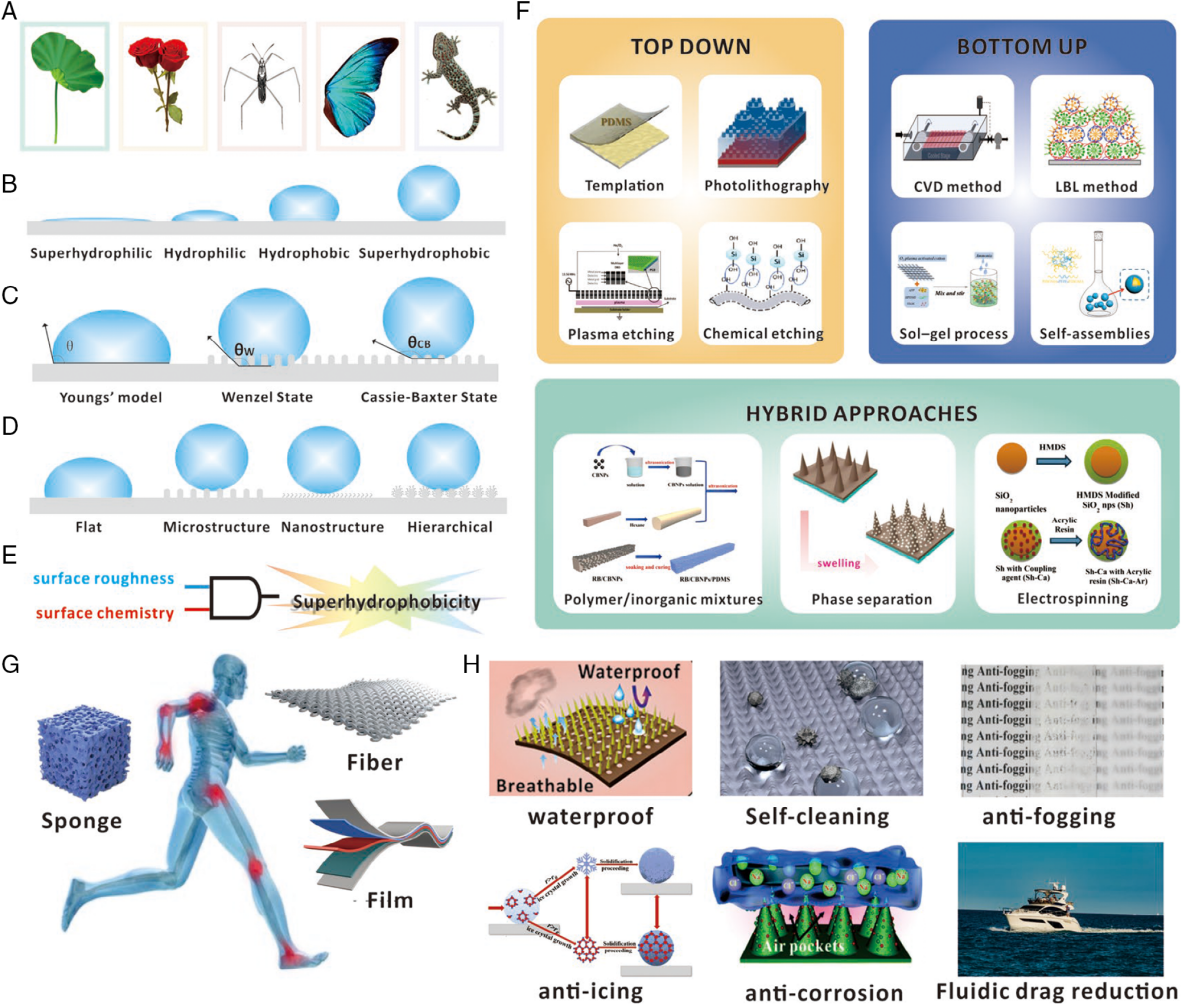
Comprehensive overview of superhydrophobicity: principles, surface fabrication, flexible structures, and application. (A) Superhydrophobicity in nature, including lotus leaf, rose petals, water striders, butterfly wings, and geckos. (B) Various phenomena of droplets on different solid surface with different wetting properties. (C) Schematics of three wetting models. (D) Droplet behaviors on solid surface with different surface structures. (E) Two key factors of superhydrophobicity, the surface roughness and surface chemistry. (F) Three basic methods to fabricate superhydrophobic surface, including top‐down (in yellow), bottom‐up (in blue), and hybrid approaches (in green). Reproduced with permission.^[^ ^64^ ^]^ Copyright 2019, American Chemical Society. Reproduced with permission.^[^ ^65^ ^]^ Copyright 2017, Wiley‐VCH. Reproduced with permission.^[^ ^66^ ^]^ Copyright 2019, American Chemical Society. Reproduced with permission.^[^ ^67^ ^]^ Copyright 2020, Elsevier. Reproduced with permission.^[^ ^68^ ^]^ Copyright 2016, American Chemical Society. Reproduced with permission.^[^ ^69^ ^]^ Copyright 2019, Elsevier. Reproduced with permission.^[^ ^70^ ^]^ Copyright 2020, Elsevier. Reproduced with permission.^[^ ^71^ ^]^ Copyright 2019, Elsevier. Reproduced with permission.^[^ ^72^ ^]^ Copyright 2020, Elsevier. (G) Common structures of superhydrophobic wearable sensors, the sponges, films and fibers. (H) Applications and merits of superhydrophobicity. Reproduced with permission.^[^ ^73^ ^]^ Copyright 2022, American Chemical Society. Reproduced with permission.^[^ ^74^ ^]^ Copyright 2016, American Chemical Society. Reproduced with permission.^[^ ^75^ ^]^ Copyright 2021, Elsevier. Reproduced with permission.^[^ ^36^ ^]^ Copyright 2021, Royal Society of Chemistry.

Superhydrophobicity, which refers to the extreme wetting effect, has been observed in various natural organisms such as plants (e.g., lotus, rose petal), insects (e.g., water strider, butterfly), and animals (e.g., birds and gecko), as shown in Figure 2A.^[^ ^76^, ^77^ ^]^ To understand the concept of superhydrophobicity, it is necessary to introduce the principle of solid material wettability briefly. The angle formed by the liquid/gas interface on a solid surface, known as the contact angle, is generated by the solid–liquid–gas three‐phase contact line and the tangent to the liquid–gas interface.^[^ ^78^, ^79^ ^]^ It can be firstly quantified by the contact angle (*θ_CA_*) of a resting droplet on the surface (Figure 2B) to evaluate the superhydrophobicity. Based on the range of contact angle values, the surface can be classified as superhydrophilic surface with the *θ_CA_* < 10°, hydrophilic surface with the 10° < *θ_CA_* < 90°, hydrophobic surface with 90° < *θ_CA_* < 150°, and superhydrophobic surface with *θ_CA_* > 150°.^[^ ^80^ ^]^ Secondly, it is necessary for water droplets to exhibit ease of rolling off the surface rather than adhering to it. The ease of droplet rolling can be described by the contact angle hysteresis of a surface, which is defined as the difference between the maximum (advancing) and minimum (receding) stable contact angle.^[^ ^81^, ^82^ ^]^ Young's equation is commonly used to describe the relationship between contact angle and surface tension at solid, liquid, and vapor interfaces.^[^ ^83^ ^]^ This equation is typically applied to perfectly flat surfaces and concludes that the wettability of a surface is determined by its surface free energy (Figure 2C):

$$\;\gamma_{SG}=\gamma_{SL}+\gamma_{LG}\cos\theta$$

where *γ_SG_*, *γ_SL_*, and *γ_LG_* represent the surface tension at the solid–gas, solid–liquid, and liquid–gas interfaces. In reality, a completely smooth and chemically homogeneous surface is rarely found, hence other factors besides surface free energy play an important role in determining the wetting behavior of solid surfaces. Among these factors, topological roughness has been found to be a crucial determinant. To explain the relationship between surface roughness and wettability, two models, the Wenzel model and the Cassie‐Baxter model, have been proposed as shown in Figure 2D.^[^ ^84^, ^85^ ^]^ The former model predicts that increasing roughness leads to an increase in the apparent contact angle, while the latter model proposes that the apparent contact angle is determined by the fraction of the droplet in contact with the solid surface and the fraction in contact with air, which is influenced by the roughness and chemical composition of the surface. Specifically, in the Wenzel model, the complete wetting of a rough surface by a liquid is assumed. The model defines surface roughness, represented by the ratio of the actual surface area in contact with the liquid to its projection on the horizontal plane, as a crucial factor in determining the wetting behavior of a surface. This relationship can be described mathematically as follows:

$$\cos\;\theta_{\gamma }=\gamma \cos\theta$$

where *γ* refers to the roughness of the surface, and *θ_γ_* is the apparent contact angle. The roughness ratio is always greater than 1, as a perfectly smooth surface has a ratio of 1. Therefore, surface roughness can enhance the inherent wettability of a surface, regardless of whether it is initially hydrophobic or hydrophilic.^[^ ^86^ ^]^ Wenzel's model predicts a fully wetted state where the droplet cannot slide on the surface. Thus, it fails to describe how water droplets slide off the surface when the surface energy *γ* is much larger than 1. In contrast, the Cassie–Baxter model assumes a smaller solid‐liquid contact area due to the presence of air pockets underneath the rough surface.^[^ ^87^ ^]^ The effect of the solid–air composite interface on the surface contact angle can be determined using the following equation:

$$\cos\;\theta_{\gamma }=f_{1}\;\cos\theta_{1}+f_{2}\cos\theta_{2}$$

where *θ*_1_ and *θ*_2_ are apparent contact angle of the droplet, *f*_1_ and *f*_2_ are the ratio of the solid–liquid contact area and the liquid–gas ratio on the rough surface, respectively (here, *f*_1_ + *f*_2_ = 1).^[^ ^37^ ^]^ In this model, it is assumed that the droplet contacts the rough top without penetrating the valley, and the contact angle between air and water varies by a maximum of 180°, *θ*_2_ = 180°, then have:

$$\cos\;\theta_{\gamma }=f_{1}\left(\cos\theta_{1}+1\right)-1$$

The Cassie–Baxter model provides a framework to understand the mechanism behind the well‐known “Lotus effect,” achieving by the combination of hierarchical micro‐nanostructures (randomly distributed micro‐capillary nanostructures), and low surface energy coatings (skin wax materials), which result in self‐cleaning properties. Hence, the combination of hierarchical micro‐nanostructures and low surface energy coatings acts as two essential factors that determine the superhydrophobicity of a surface. These factors operate as an AND gate, as illustrated in Figure 2E, resulting in various structures(Figure 2G) and applications (Figure 2H).^[^ ^88^ ^]^

### 2.2 Progress in design strategies of flexible superhydrophobicity

Wearable electronics, by refining the rigidness, bulkiness, and limited portability inherent to conventional sensors, is urging specific demands from incorporated materials to fabrication methodologies. Flexible substrates, for example, sponge‐based materials, stretchable films, and fibers, have been extensively reported not only as platforms for flexible superhydrophobic surfaces but also as vital carriers for wearable electronics. This provides the opportunity to combine the surface superhydrophobicity with flexible electronics for functionality exploration. Thanks to the intrinsic properties of such materials, these wearable systems can be optimized to exhibit multifunctionality, high sensitivity, rapid response, and cost‐effectiveness for diverse applications. To achieve the aim of superhydrophobicity on flexible substrates, the hierarchical structure is normally required to serve as a solid–liquid interface, which helps to disperse the accumulation of peak stress and prevent the collapse of micro‐nano structures. However, flexible substrates are also more susceptible to superhydrophobic failure due to the anti‐wetting performance under various deformations. During the dynamic processes, this issue becomes more prominent. This is principally attributed to the degradation of surface structures throughout the deformation process, the consequential detachment of functional materials and the wetting phenomena due to the alterations in scale of surface roughness. Consequently, additional challenges need to be considered for flexible wearable electronics with superhydrophobic functions. The development of superhydrophobic surfaces requires both surface roughness and low surface energy and can be categorized into two primary types: top‐down^[^ ^32^, ^89^, ^90^ ^]^ and bottom‐up.^[^ ^31^, ^91^, ^92^ ^]^ The top‐down strategies create micro/nanostructures on an existing hydrophobic surface, utilizing techniques such as templating, photolithography, plasma etching, and chemical etching. Such methods typically involve bulk substrates that offer higher mechanical durability and robustness but have lower throughput and higher costs. In contrast, bottom‐up strategies involve applying low surface energy materials to a target surface, such as through CVD,^[^ ^93^ ^]^ LBL^[^ ^94^ ^]^ deposition, sol–gel method,^[^ ^95^ ^]^ and self‐assembly.^[^ ^96^ ^]^ While these methods may be less stable, they offer a greater variety of structural possibilities and additional functionalities such as superamphiphobicity, transparency, and photoheating. Hybrid techniques that combine top‐down and bottom‐up strategies can produce composite rough surfaces with hierarchical roughness, providing a wider range of properties and potential applications. Through the implementation of these strategies, superhydrophobic surfaces can be achieved on a variety of substrates, including elastomers (blocks, and films),^[^ ^97^ ^]^ textiles,^[^ ^77^ ^]^ and even paper based substrate,^[^ ^98^ ^]^ among others (Figure 2F).

#### 2.2.1 Sponges

**FIGURE 3:**
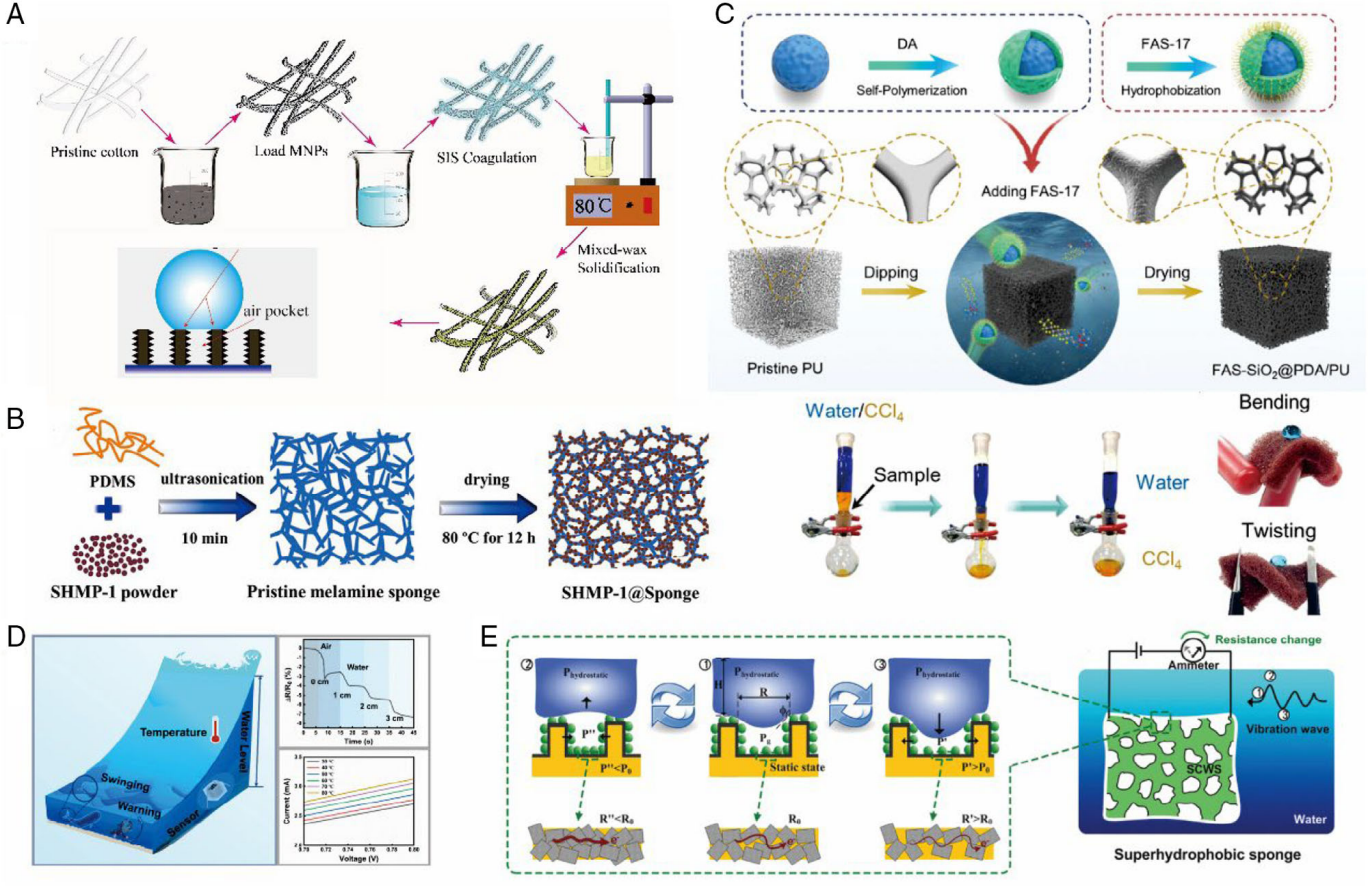
Common surface modification strategies employed for sponge structures and sensing applications. Specifically, (A) synthesis of superhydrophobic sponges. Reproduced with permission.^[^ ^99^ ^]^ Copyright 2019, Elsevier. (B) Depicts the process schematic of a melamine skeleton sponge with polymer SHMP‐1 powder. Reproduced with permission.^[^ ^100^ ^]^ Copyright 2022, Elsevier. And (C) showcases a fluorination strategy to produce a superhydrophobic PU sponge, along with its schematic representation and oil‐water separation performance evaluation. Reproduced with permission.^[^ ^101^ ^]^ Copyright 2023, Wiley‐VCH. Furthermore, (D) demonstrates the development of a temperature and pressure sensor based on a superhydrophobic sponge. Reproduced with permission.^[^ ^29^ ^]^ Copyright 2021, American Chemical Society. (E) A tiny vibration sensing superhydrophobic sponge based on Cassie state, designed to avoid crosstalk. Reproduced with permission.^[^ ^102^ ^]^ Copyright 2018, Wiley‐VCH.

Flexible porous sponge materials can be generated using a variety of techniques, typically using elastomers as the backbone with desired substrate roughness through template synthesis, vapor deposition, self‐assembly, and phase separation strategies.^[^ ^103^ ^]^ Various materials can be used for the sponge skeleton, including thermoplastics,^[^ ^104^ ^]^ thermosets,^[^ ^105^ ^]^ elastomers,^[^ ^106^ ^]^ fluorinated polymers,^[^ ^107^ ^]^ conductive polymers,^[^ ^108^ ^]^ organosilanes,^[^ ^109^ ^]^ long alkyl chain compounds, and hydrophobic carbon‐based materials.^[^ ^110^ ^]^ To achieve surface roughness, different particles such as carbon nanoparticles,^[^ ^111^ ^]^ metal nanoparticles,^[^ ^112^ ^]^ and inorganic non‐metallic nanoparticles^[^ ^113^ ^]^ can be introduced to the matrix. Dip coating is the most widely used method, with the merits of easy operation and timesaving.^[^ ^114^ ^]^ For example, Liu et al. used a non‐polluting dip‐coating technique to prepare superhydrophobic cotton‐based sponges with controllable surface morphology and wetting properties, achieving a contact angle of 162° and a rolling angle smaller than 5° through the hierarchical absorbent regulation as shown in Figure 3A.^[^ ^99^ ^]^ Similarly, Gong and colleagues efficiently combined PDMS and cross‐linked microporous polymers on the surface of melamine sponge based on the dip‐coating method (Figure 3B).^[^ ^100^ ^]^ Zhen et al. reported a HIDS@PDA core‐shell particle of SiO_2_ self‐assembled on PU sponge with extremely strong robustness. The proposed superhydrophobic sponges exhibit potentials for water–oil separation which can effectively manipulate liquids at the liquid/air/solid interface as demonstrated in Figure 3C.^[^ ^101^ ^]^ Furthermore, these superhydrophobic processing techniques do not compromise the inherent properties of the sponge structure itself, which typically consists of interconnected pores, offering characteristics such as lightweightness, high absorbency, and large surface area. These attributes endow itself with potential application value in the realm of wearable sensors. For instance, its compressibility enables utilization in various sensing modes such as resistive‐based pressure sensing, friction‐based nanogenerators, and piezoelectric mechanisms, facilitating measurements of pressure, stretching, vibrations (acoustic signals), and other strain‐related behaviors.^[^ ^29^, ^102^, ^115^ ^]^ The porous structures also render the possibility of chemical sensing applications like humidity and gas sensing. Moreover, it can simultaneously enhance the sensor's breathability, flexibility, and comfort due to its lightweight nature (Figure 3D,E).

#### 2.2.2 Stretchable films

**FIGURE 4:**
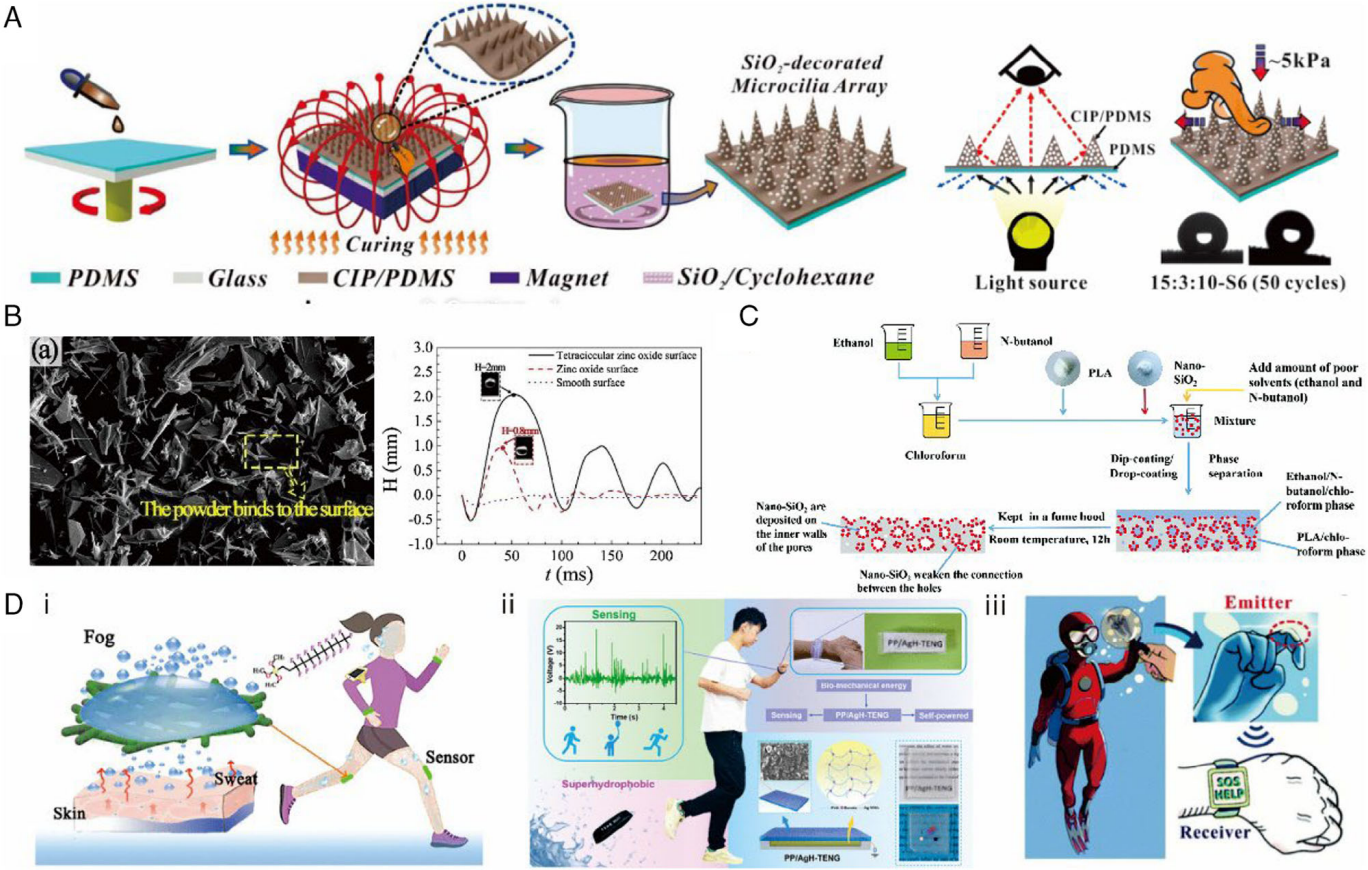
Fabrication methods for superhydrophobicity on flexible sponge substrates. (A) The preparation of transparent superhydrophobic PDMS films with magnetic field‐assisted microcilia structures. Reproduced with permission.^[^ ^116^ ^]^ Copyright 2020, Wiley‐VCH. (B) Droplet rebound capability of superhydrophobic films via powder scattering technology. Reproduced with permission.^[^ ^117^ ^]^ Copyright 2020, Elsevier. (C) Superhydrophobic PLA films produced using a phase separation method. Reproduced with permission.^[^ ^118^ ^]^ Copyright 2019, Royal Society of Chemistry. (D) Potential applications based on these superhydrophobic films include (i) anti‐interference strain sensors. Reproduced with permission.^[^ ^28^ ^]^ Copyright 2023, American Chemical Society. (ii) Humid‐resistance self‐powered TENG sensors. Reproduced with permission.^[^ ^119^ ^]^ Copyright 2022, American Chemical Society. And, (iii) underwater strain sensors with superhydrophobic surfaces serving as insulation barriers. Reproduced with permission.^[^ ^36^ ^]^ Copyright 2021, Royal Society of Chemistry.

Stretchable superhydrophobic thin films exhibit significant potential in flexible electronic applications, offering the capability to effectively reduce interference and provide a stable working environment for various scenarios. On the other hand, the emergence of communication and computation modules adapted to sensors, including technologies such as flexible circuits and antennas, has further expanded the demand for superhydrophobic and stretchable thin film materials. The incorporation of superhydrophobic technology can introduce additional protective layers, enhancing the durability of these electronic materials in harsh environments.^[^ ^28^, ^36^ ^]^ Flexible superhydrophobic thin films can be based on a variety of substrates, including metals,^[^ ^120^ ^]^ silicon‐based thermoplastic elastomers,^[^ ^116^ ^]^ and thermosetting elastomers.^[^ ^121^ ^]^ Such substrates are typically thin, stretchable, and flexible, making them suitable for adapting to complex or irregular surfaces. These films are generally prepared using integrated surface technologies such as the template method,^[^ ^122^ ^]^ blade coating,^[^ ^123^ ^]^ spray coating,^[^ ^124^ ^]^ spin coating,^[^ ^125^ ^]^ drop casting,^[^ ^126^ ^]^ printing,^[^ ^127^ ^]^ electrospinning,^[^ ^128^ ^]^ and phase separation.^[^ ^118^ ^]^ These methods result in strong and robust films with a superhydrophobic surface. For instance, Dai and colleagues developed a microcilia array based on spin‐coated liquid‐exclusion‐induced phase separation (Figure 4A). The resulting film exhibited wear resistance and transparency, showcasing its potential application as a self‐cleaning protective film for touchscreens.^[^ ^116^ ^]^ Zhou et al. utilized powder scattering to create a superhydrophobic zinc oxide/silicone rubber composite material, demonstrating excellent droplet rebound performance as shown in Figure 4B.^[^ ^117^ ^]^ Zhong et al. induced phase separation in PLA/SiO_2_ composite films, resulting in highly non‐toxic films suitable for the transport of medical particles (Figure 4C).^[^ ^118^ ^]^ For wearable sensors based on superhydrophobic thin films, the low surface energy and roughness can be achieved using nanoscale particles made of sensor‐active materials. For instance, for common resistive and TENG‐based sensors, the superhydrophobic properties can enable the creation of encapsulation‐free electronic sensor devices (Figure 4D).^[^ ^119^, ^129^ ^]^

#### 2.2.3 Fibers

**FIGURE 5:**
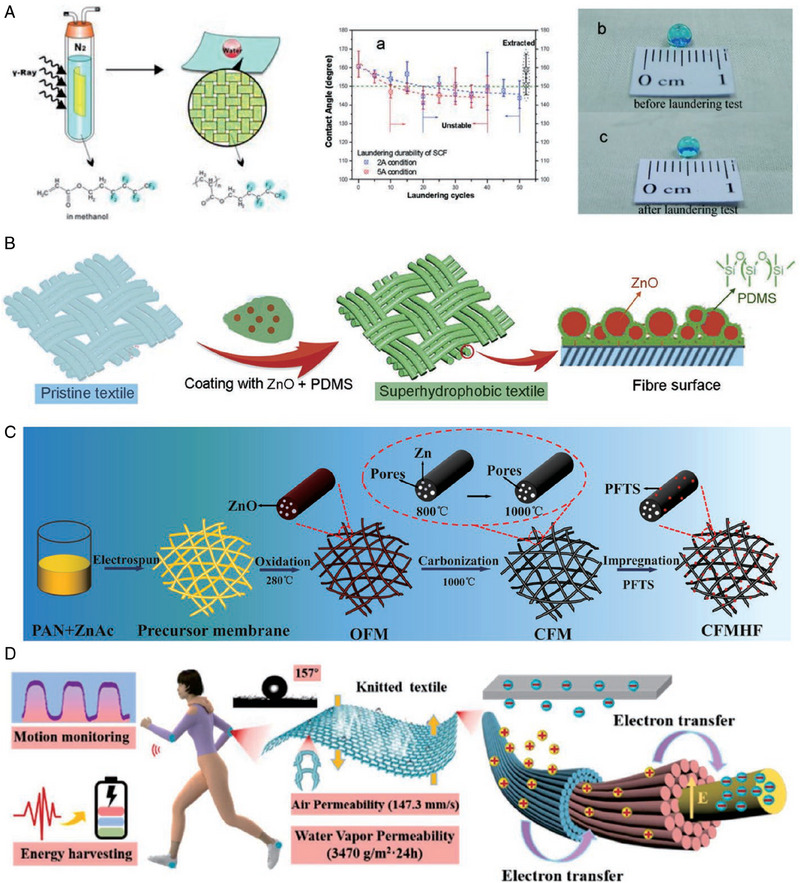
Various methods for achieving superhydrophobic surfaces on fabrics and their potential applications. (A) Schematic illustration of the radiation‐induced graft polymerization approach to construct superhydrophobic surfaces on cotton fabrics that remain stable after commercial laundering. Reproduced with permission.^[^ ^130^ ^]^ Copyright 2010, Wiley‐VCH. (B) Schematic of the preparation of a ZnO‐PDMS‐coated fabric with mechanical stability, UV durability, and UV shielding. Reproduced with permission.^[^ ^131^ ^]^ Copyright 2018, Elsevier. (C) A carbon fiber superhydrophobic membrane prepared using the electrospinning carbonization method. Reproduced with permission.^[^ ^128^ ^]^ Copyright 2021, Elsevier. (D) Showcases the promising use of superhydrophobic fabrics for energy harvesting and wearable sensing. Reproduced with permission.^[^ ^132^ ^]^ Copyright 2022, Elsevier.

Superhydrophobic fiber‐based surfaces have numerous applications in fields such as self‐cleaning textiles,^[^ ^133^ ^]^ waterproof clothing,^[^ ^130^ ^]^ and antibacterial textiles.^[^ ^134^ ^]^ In the last decade, there has been extensive research on achieving superhydrophobicity on fibrous substrates, particularly on fabric surfaces that have natural micron‐scale roughness from the fibers themselves and the woven structure.^[^ ^135^ ^]^ Secondary nanoscale structures have been generated using nanoparticle deposition, polymer grafting, electrospinning, and other strategies to achieve superhydrophobicity. However, weak adhesion between the incorporated particles and the fibrous substrate has been an important issue. To address this, Deng et al. synthesized wash‐durable superhydrophobic fabric by simultaneously inducing grafting of fluorinated acrylates onto cotton fibers using synchrotron radiation (Figure 5A).^[^ ^130^ ^]^ Ren et al. used linked PDMS and ZnO nanoparticles as coating materials to prepare a superhydrophobic fabric with UV protection (Figure 5B).^[^ ^131^ ^]^ Beyond surface modification of traditional fabrics, novel substrates created through electrospinning have also been explored as self‐cleaning superhydrophobic materials. Sun et al. devised a method utilizing polyacrylonitrile (PAN) as a precursor to produce carbon fiber membranes through electrospinning and subsequent carbonization, resulting in substrates with exceptional stability in harsh environments (Figure 5C).^[^ ^128^ ^]^ As fabrics have various application scenarios, researchers are focused on multi‐functionality, leading to smart fabrics with waterproofing, radiation protection, thermal management, and health monitoring functions. Take Figure 5D as an example, these superhydrophobic fabrics also serve as platforms for wearable sensing systems.^[^ ^132^ ^]^ Recent reports based on fiber materials include the triboelectric nanogenerators, piezoelectric nanogenerators, resistive and capacitive strain sensors, demonstrating wide application spectrum in our daily life.

### 2.3 Potentials of superhydrophobic surfaces in flexible electronics

Reports on these flexible superhydrophobic surfaces, endowed with excellent stretchability and conformity, aim to resolve the instability caused by the fragile structure and impermanent superhydrophobicity. In the domain of wearable electronics, such functional surfaces enable inherently unstable devices to participate stably in daily human activities in an encapsulation‐free manner, even in the face of dual environmental challenges from both ambient conditions and attachment points. Superhydrophobic surfaces hold tremendous potential for applications in numerous fields that require clean surfaces, such as solar panels, owing to their self‐cleaning properties.^[^ ^136^, ^137^ ^]^ They can prevent water film formation, minimize the risk of scattering issues in optical applications, and reduce the likelihood of surface ice attachment. Additionally, the air cushion formed by the reduced contact area can enhance corrosion resistance by averting the impact of water, moisture, oxygen, and other effects on the surface. Superhydrophobic surfaces can also function as an insulating layer in an electrolyte environment to safeguard against short circuits. Moreover, these surfaces possess the potential for drag reduction and anti‐biofouling in underwater environments.^[^ ^138^ ^]^ To attain high‐durability and stable devices, superhydrophobic flexible electronics aims to combine the benefits mentioned above. However, depending on the application scenario, additional requirements may be necessary. Specifically, flexibility and stretchability are essential for wearable sensors that require imperceptible signal collection based on the existing design principles and application scope of superhydrophobic surfaces. Therefore, it is crucial to maintain the superhydrophobicity of the surface when exposed to various environmental interferences, ensuring long‐term working stability and robustness. In the following section, we review the superhydrophobic design strategies and outcomes for various wearable sensors to elucidate their cutting‐edge potential for diverse application scenarios such as healthcare monitoring, motion indicator and human–computer interactions.

## 3 COUPLING OF FLEXIBLE ELECTRONICS WITH SUPERHYDROPHOBICITY

**FIGURE 6:**
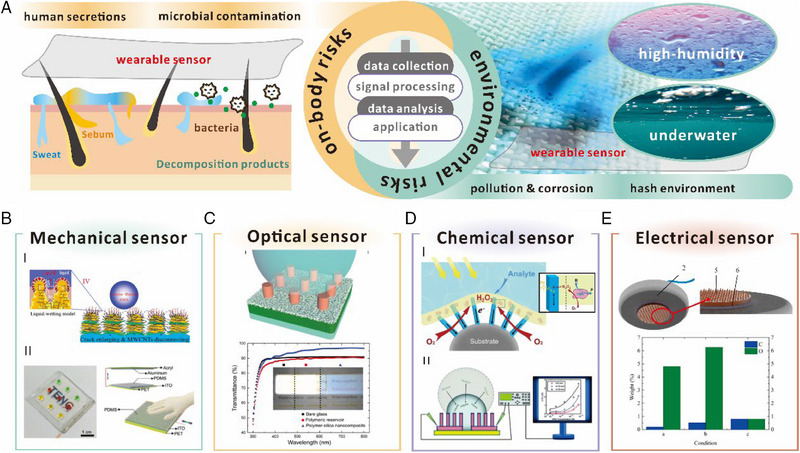
Classification and performance advantages of superhydrophobic wearable sensors. (A) Schematic illustration of the double risk faced by wearable sensors with superhydrophobic surfaces. The potential reasons for the degradation or failure of these sensors include pollution, corrosion, and special environments such as underwater and high humidity. Additionally, human secretions and microorganisms from the skin interface can also pose a risk. Typical wearable sensors including (B) mechanical sensors. Reproduced with permission.^[^ ^37^ ^]^ Copyright 2020, Wiley‐VCH. Reproduced with permission.^[^ ^139^ ^]^ Copyright 2019, Elsevier. (C) Optical sensors. Reproduced with permission.^[^ ^140^ ^]^ Copyright 2020, Wiley‐VCH. (D) Chemical sensors. Reproduced with permission.^[^ ^141^ ^]^ Copyright 2018, Wiley‐VCH. Reproduced with permission.^[^ ^142^ ^]^ Copyright 2013, Royal Society of Chemistry. (E) Electrical sensors. Reproduced with permission.^[^ ^143^ ^]^ Copyright 2016, Elsevier.

Wearable sensors have emerged as a promising technology for health monitoring, sports behavior analysis, and human–computer interaction interfaces.^[^ ^144^ ^]^ Compared to traditional bulk wearable systems, compact flexible wearable devices offer the superiorities such as high sensitivity, non‐intrusiveness, and user‐friendliness.^[^ ^145^ ^]^ These sensors can continuously monitor signals with a high signal‐to‐noise ratio from the sensing site. The latest wearable sensor devices incorporate stretchable films based on the human skin as the coupling site. The skin provides a rich source of information about a wide range of human characteristics, including blood pressure,^[^ ^146^ ^]^ blood sugar,^[^ ^147^ ^]^ blood oxygen,^[^ ^148^ ^]^ heart rate,^[^ ^149^ ^]^ temperature,^[^ ^150^ ^]^ exercise behavior,^[^ ^151^ ^]^ and action‐based disease information.^[^ ^152^, ^153^ ^]^ Thus, in order to achieve comprehensive sensing capabilities in wearable sensors, they are often designed to have a modulus that can couple with the skin. However, long‐term monitoring of such information inevitably exposes the sensor to both internal and external risks. External risks include active and passive cleaning, uncontrollable weather, and environmental factors (such as changes in humidity or exposure to underwater environments), and environmental corrosion. All of above conditions can potentially cause the performance decay of the sensing system owing to the degradation of the active sensing or substrate materials in long‐term real‐world applications. On the other hand, as shown in Figure 6A, for the inner side of the wearable sensor that is close to the skin, several factors including chemical corrosion, biological adhesion, and mechanical noise can impact the sensor's performance. This risk is amplified due to the skin's role as a barrier and its constant respiration. Bacterial populations as high as 10 billion per square centimeter have been observed on the skin, which secrete proteins and cellular waste products that can interfere with sensor readings.^[^ ^154^ ^]^ In addition, various secretions on the skin's surface, including sweat, introduce minerals and organic pollutants that can continuously accumulate on the wearable sensor. The accumulation might have serious effects on the micro/nano‐structured sensing array, and in some cases, cause the sensing mechanism to fail. Thus, incorporating a superhydrophobic surface with anti‐corrosion, anti‐wetting, self‐cleaning properties, and low adhesion can significantly enhance the durability and versatility of wearable sensors for long‐term usage under various environments. In this section, we provide a comprehensive summary of existing wearable sensing devices with superhydrophobic surfaces, specifically mechanical, electronic, chemical, and optical devices (Table 1). In this table, we focus on the key design goals, considerations, and challenges associated with superhydrophobic surfaces that serve as the signal source and target application of interest for four types of wearable sensors in Figure 6B–E. The typical applications, processing strategies, and sensing performance of such superhydrophobic sensors are summarized in the table. Additionally, we emphasized the special functions of these superhydrophobic surfaces, which is mainly contributed by selection of specific materials with elaborate design of surface architectures.

**TABLE 1:** A comprehensive review of wearable sensors with superhydrophobic surfaces: Classifications, materials, fabrication methods, purpose of superhydrophobic surface and promising applications.

Sensor types	Materials	Fabrication methods	Purpose of superhydrophobicity	Potential applications	Sensing performances	Ref.
Piezoresistive strain sensor	CNT_1_, FAS_2_, PDMS_3_,	Template method, graft copolymerization	Conductivity, anti‐corrosion, self‐cleaning, anti‐liquid jet	Motion sensor	Gauge factor 22.64; Working range: 200%; 10,000 stretching–relaxing cycles	[60]
Piezoresistive strain sensor	MWCNT/G_4_, PDMS	Spraying coating	Conductivity, anti‐liquid interference, anti‐corrosion, anti‐bio‐adhesion, self‐cleaning	Motion sensor	Gauge factor 1989; working range 170%; response time 150 ms; 1000 stretch‐release cycles	[37]
Piezoresistive strain sensor	CB_5_, silica, PDMS	Self‐assembly, dip coating	Anti‐conductive solution interference, anti‐corrosion, anti‐liquid jet, self‐cleaning	Motion sensor, signal generator	Gauge factor 354; working range 250%;10,000 stretch‐release cycles	[36]
Piezoresistive strain sensor	GO_6_/CS_7_, silica, PDMS, PSKF_8_	Dip coating	Water repellency, self‐cleaning, anti‐pollution, stability in extreme cold condition	Motion sensor, signal generator, thermal management	Gauge factor: *x* direction −1.06, *y* direction −2.08; working range 60%; response time 22 ms; 4000 stretch‐release cycles	[155]
Piezoresistive strain sensor	rGO_9_/PFDT_10_/PDA_11_/PU_12_	Electrospinning, dip coating	Anti‐corrosion, water repellency	Motion sensor	Gauge factor 221; working range 590%; 10,000 stretch‐release cycles	[156]
Piezoresistive strain sensor	MXene, candle ash, paper	Dip coating, deposition method	Anti‐corrosion, self‐cleaning, water repellency	Motion sensor, arrayed device	Gauge factor: 17.4; detection limit 0.1%; 1000 stretch‐release cycles	[157]
Piezoresistive strain sensor	rGO, PDMS	Prestretch‐based deposition and ion sputtering	Conductivity, anti‐corrosion, water repellency	Motion sensor	Gauge factor 665.6; working range 300%; detection limit 0.1%; 10,000 stretch‐release cycles	[158]
Piezoresistive strain sensor	GO, silica, TPU_13_	Electrospinning, dip coating	Anti‐corrosion, water repellency	Motion sensor	Gauge factor 5.9; working range 400%; 600 stretch‐release cycles	[159]
Piezoresistive strain sensor	AgNW_14_/ACNT_15_, PDMS, TPU	Electrospinning, dip coating, self‐assembly	Water repellency, self‐cleaning, anti‐corrosion	Motion sensor, health‐care monitor	Gauge factor 1.36E5; working range 100%; conductivity 3506.8 S/m; 1200 stretch‐release cycles	[160]
Piezoresistive strain sensor	AgNPs/MWCNTs, silica, paper	Dip coating, spraying method	Water repellency, self‐cleaning, anti‐corrosion, anti‐liquid jet	Water droplet‐induced vibration sensor	Gauge factor 263.34; resolution 0.098%; response time 78 ms; 12,000 stretch‐release cycles	[161]
Piezoresistive strain sensor	P(AAm‐*co*‐HEMA)_16_‐MXene‐AgNPs hydrogel	Dip coating	Water repellency, anti‐corrosion, anti‐liquid jet, anti‐bacteria,	Motion sensor	Gauge factor 4.08; working range 120%; response time 120 ms; 400 stretch‐release cycles	[162]
Piezoresistive strain sensor	AgNPs_17_, PFDT, RB_18_	Dip coating, self‐assembly	Water repellency, anti‐liquid interference, anti‐corrosion, anti‐liquid jet, anti‐bacteria	Motion sensor	Gauge factor 10E8; working range 1000%; 1000 stretch‐release cycles	[163]
Piezoresistive strain sensor	P(AA‐*co*‐AAm)_19_/graphene hydrogel	Sol–gel method	Water repellency, anti‐liquid jet, self‐cleaning	Motion sensor	Gauge factor 9.97; working range 1000%; 10,000 stretch‐release cycles	[164]
Piezoresistive strain sensor	CNT, PDMS,	Sol–gel method, template method, laser irradiation	Water repellency, anti‐corrosion	Motion sensor	Gauge factor 3.1; working range 100%; 5000 stretch‐release cycles	[165]
Piezoresistive strain sensor	AgNPs, STA_20_, PDMS,	Dip‐coating	Water repellency, anti‐corrosion, Joule heating performance	Motion sensor	Gauge factor 3.1; working range 00%; conductivity 102 S cm^−1^; 1000 stretch‐release cycles	[166]
Piezoresistive pressure sensor	F‐rGO_21_@CNTs/CS aerogel	Freeze‐drying and dip‐coating	Water repellency, anti‐liquid interference, anti‐corrosion	Motion sensor	Sensitivity 4.97 kPa^−1^; response time 170 ms; 1000 loading–unloading cycles	[167]
Piezoresistive pressure sensor	P‐rGO_22_@WS_23_	Template method, self‐assembly	Water repellency, anti‐liquid interference, anti‐corrosion	Motion sensor	Sensitivity 4.93 kPa^−1^; working range 0–5 kPa; response time 160 ms; 1000 loading–unloading cycles	[168]
Piezoresistive pressure sensor	MXene@c‐CNT/CCS_24_ FAS aerogel	Freeze‐drying, CVD	Water repellency, anti‐corrosion	Motion sensor, arrayed device	Sensitivity 3.84 kPa^−1^; working range 0–80 kPa; response time 62 ms; 200 loading–unloading cycles	[169]
Piezoresistive pressure sensor	PPy_25_/MXene cotton fabric	Dip‐coating	Water repellency, anti‐corrosion, self‐cleaning, anti‐liquid interference	Motion sensor	Sensitivity 20.1 kPa^−1^; working range 0–80 kPa; response time 80 ms;	[45]
Piezoresistive pressure sensor	CNT/CB, PDMS	Dip‐coating	Water repellency, anti‐corrosion, self‐cleaning, anti‐liquid interference	Motion sensor, signal generator	Gauge factor 7.747, detection limit 0.04%; sensitivity 0.0198 kPa^−1^; working range 0–200 kPa; 10,000 cycles	[73]
Capacitive sensor	Nickel surfaces and PPFC_26_ coating	Photolithography	Water repellency, superoleophilic	Micro‐oil detector	Detection limits below oil films of 1.5 µm or smaller	^[^ ^170^ ^]^
Piezoelectric nanogenerators based sensor	PVT_27_, CNT, P(PFDA‐*co*‐EGDA)_28_	iCVD	Water repellency, anti‐corrosion, self‐cleaning, anti‐bacteria, anti‐pollution, anti‐interference	Motion sensor, breath monitor	Sensitivity 0.35 V kPa^−1^; detection range 5–200 g	[171]
Triboelectric nanogenerator based sensor	Au, FAS, PET_29_,	Lithography, metal bonding	Water repellency, self‐cleaning, anti‐pollution, anti‐liquid interference	Self‐powered breathing detector for humid environments	Open circuit voltage 48 V; 50,000 contact‐release cycles	[172]
Triboelectric nanogenerator based sensor	Epoxy resin, PDMS, PTFE_30_, Cu, PFOTES_31_	Template method, sol–gel method	Water repellency, self‐cleaning, anti‐pollution, anti‐liquid interference	Humidity‐resistant energy harvester and motion sensor	Open circuit voltage 21.6 V, charge transfer 10 nC; output power 16 µW	[173]
Triboelectric nanogenerator based sensor	PDMS, ITO_32_, PET	Lithography, self‐assemble	Water repellency, anti‐humidity interference	Humidity‐resistant energy harvester	Open circuit voltage 400 V; current density 17 µA cm^−2^	[139]
Triboelectric nanogenerator based sensor	PFTS_33_, m‐BN_34_/PVT	Sol–gel method	Anti‐humidity interference	Humidity‐resistant energy harvester, humidity sensor, and motion sensor	Output power 43 W m^−2^; sensiticity 80 nA RH^−1^; response time 0.6 ms	[17]
Triboelectric nanogenerator based sensor	BH‐PDMS_35_, Au	Template method	Water repellency, anti‐pollution, anti‐humidity interference	Sweat‐resistant self‐powered motion sensor	Open circuit voltage 60 V; short circuit current 150 nA; response time 0.6 ms; 10,000 contact‐release cycles	[174]
Triboelectric nanogenerator based sensor	PTFE, ZnO, Al	Self‐assemble	Water repellency, droplet rebound	Droplet‐induced energy harvesting	Open circuit voltage 1.4 V; short circuit current 1.3 µA	[175]
Triboelectric nanogenerator based sensor	Silica, tape, Cu	Dip‐coating	Water repellency, super hemophobia, anti‐bacteria, anti‐pollution	Smart intravenous infusion monitor, smart blood transfusion monitor	1.97 nA output current for 2.28 mL droplet	[176]
Triboelectric nanogenerator based sensor	HDFS_36_, Al	Chemical etching	Water repellency, droplet rebound	Droplet‐induced energy harvesting and rebound behavior	Short circuit voltage 150.5 V; induced charge 3.76 nC for water droplet	[177]
Triboelectric nanogenerator based sensor	SiO_2_/P(VDF‐TrFE)_37_	Electrospinning, Stretching‐transferring‐relaxing and shaping (STRS) process	Water repellency, self‐cleaning, droplet rebound	Water‐jet induced energy harvesting	At a water flow rate of 11 mL s^−1^, output voltage 36 V; output current is 10 µA	[178]
Triboelectric nanogenerator based sensor	Mg‐Al LDHs_38_ PFTS,	Self‐assemble, CVD	Water repellency, droplet rebound, anti‐corrosion,	Droplet‐induced energy harvesting	Output voltage 13 V, current density 1.6 µA cm^−2^	[179]
Triboelectric nanogenerator based sensor	CNF_39_, FEP_40_	Self‐assemble	Water repellency, droplet rebound, anti‐corrosion, long‐term stability	Water wave monitor	Output voltage 120 V; 10,000 cycles	[180]
Optical sensor	Silica NPs, PDMS, CIP	Self‐assemble, dip‐coating	Water repellency, transparency, self‐cleaning, droplet rebound, anti‐corrosion, long‐term stability	Touch screen protector	Optical transparency	[116]
Optical sensor	Octyltrimethoxysilane	Dip‐coating	Water repellency, transparency, self‐cleaning	Optical sensor protector	Optical transparency	[181]
Optical sensor	Silica, PTFE	Dip‐coating	Transparency, self‐cleaning	Optical sensor protector	Optical transparency	[182]
Chemical sensor	TiO_2_, GO x_41_	Hydrothermal	Gas retention	Bio‐photoelectrochemical	Detection limit 1E‐6 m (glucose), bioassay selectivity	[141]
Chemical sensor	Pt, carbon cloth	Electrodeposition	Gas retention	Enzyme biosensor	Working range 50E‐9 m to 156E‐3 m	[183]
Chemical sensor	Nickel	Electroplating	Enrichment	Ultra‐low concentration detection	DNA detection concentration 60 am	[142]
Chemical sensor	Gold nanostructures	Self‐assemble	Water‐repellency, wetting switching method	pH sensor	Amplified pH response behavior	[184]
Gas sensor	Cr, Au, SiO_2_	Lithography, lift‐off process	Water repellency, anti‐humid interference	NO_2_ sensor	Detection limit 9.1 ppb	[185]
Gas sensor	pC7F15MAA_42_/p(DDA/PtTPP)_43_	Phase separation	Water repellency, anti‐liquid jet,	Dissolved oxygen sensor	Oxygen sensitivity 126	[186]
Gas sensor	CNF, PDMS, PU	Dip‐coating	Water repellency, anti‐corrosion, self‐cleaning	Chemical vapor sensing	Different concentrations (10, 30, 50 ppm) of organic gases are distinguished for DCM, heptane, acetone, methanol, and toluene	[187]
Electrical sensor	Cu, STA	Laser processing, dip coating	Superamphiphobicity, self‐cleaning	Potential detection	Reduce electrode interface noise	[143]

Abbreviations: 1) Carbon nanotube; 2) 1H,1H,2H,2H‐Perfluorooctyltriethoxysilane (C8F13H4Si(OCH2CH3)3; 3) Polydimethylsiloxane; 4) Multi‐walled carbon nanotubes/graphene; 5) Carbon black; 6) Graphene oxide; 7) Chitosan; 8) Polyamide/spandex knitted fabric; 9) Reduced graphene oxide; 10) 1H,1H,2H,2H‐perfluorodecane‐thiol; 11) Polydopamine; 12) Polyurethane; 13) Thermoplastic polyurethanes; 14) Silver nanowire; 15) Acid modified carbon nanotubes; 16) Polyacrylamide and hydroxyethyl methyl acrylate; 17) Silver nanoparticles; 18) Rubber band; 19) Polyacrylic acid co‐acrylamide; 20) Stearic acid; 21) 1H,1H,2H,2H‐perfluorooctyltriethoxysilane (FAS) modified reduced graphene oxide; 22) PDMS modified reduced graphene oxide; 23) Wood sponge; 24) Carboxylated carbon nanotubes (C‐CNTs)/carboxymethyl chitosan; 25) Polypyrrole; 26) Plasma polymerized fluorocarbon; 27) Poly(vinylidene fluoride‐*co*‐trifluoroethylene); 28) Poly(perfluorodecyl acrylate‐*co*‐ethylene glycol diacrylate; 29) Polyethylene terephthalate; 30) Polytetrafluoroethylene; 31) Hydrolyzed triethoxy‐1H,1H,2H,2H‐tridecafluoro‐n‐octylsilane; 32) Indiumtinoxide; 33) 1H, 1H, 2H, 2H‐Perfluorooctyltriethoxysilane; 34) Modified BN; 35) Bioinspired hierarchical PDMS; 36) Heptadecafluoro‐1,1,2,2‐tetrahydrodecyl‐trichlorosilane; 37) Poly(vinylidenelfuoride‐*co*‐trifluoroethylene); 38) Layered double hydroxides; 39) Cellulose nanofibrils; 40) Fluorinated ethylene propylene; 41) Immobilizing glucose oxidase; 42) Poly(N‐(1H, 1H‐pentadecafluorooctyl)‐methacrylamide); 43) Poly(N‐dodecylacrylamide‐*co*‐5‐[4‐(2‐methacryloyloxyethoxy‐carbonyl)phenyl]−10,15,20‐triphenylporphinato platinum(II)).

### 3.1 Wearable mechanical sensors

#### 3.1.1 Superhydrophobic piezoresistive sensor

Piezoresistive strain/pressure sensors are highly sensitive, robust, stable, and offer a high signal‐to‐noise ratio electrical response to external stimuli, including pressure, stretching, and bending. These sensors are widely regarded as the most popular technology for wearable sensing on both micro and macro scales.^[^ ^188^ ^]^ It relies on the deformation of elastic materials, which causes the material to contract in the lateral direction of elongation due to Poisson's ratio, resulting in a change in the electrical resistance of conductive materials. To improve the sensitivity, porous structure‐based pressure sensing and fracture‐based tension sensing are widely utilized. The use of flexible piezoresistive sensors in various external environments and the human body presents several challenges, as these sensors are typically thin films or patches. These challenges include molecular chain breakage, decreased working‐range, increased brittleness, and fluctuations in conductivity due to external factors such as acidity, alkalinity, salt, and humidity that can exacerbate these issues.

**FIGURE 7:**
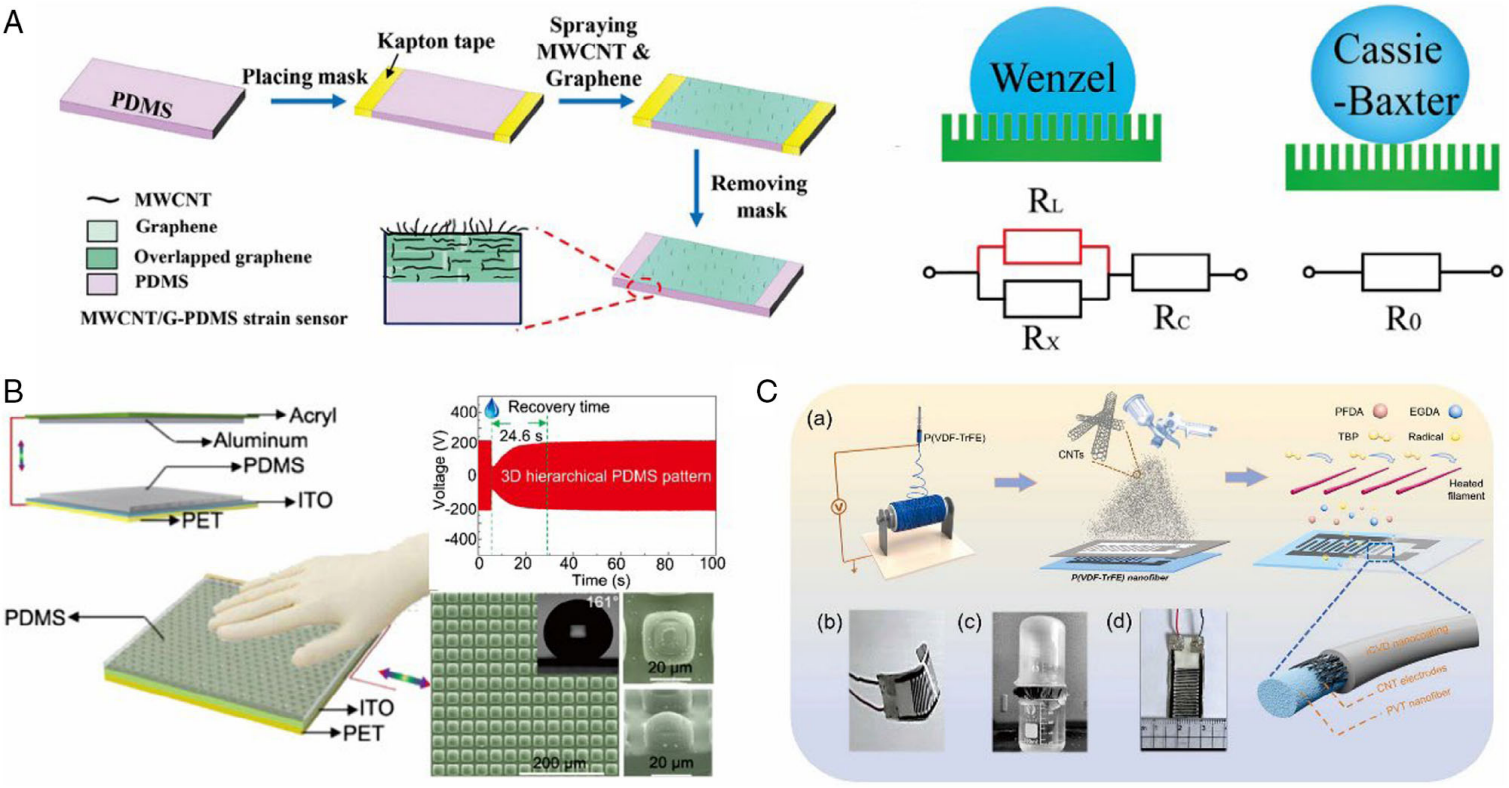
Fabrication procedures for developing mechanical sensors. Specifically, (A) illustrates a schematic of the procedure for fabricating a liquid‐interfering MWCNT/G‐PDMS strain sensor. Reproduced with permission.^[^ ^37^ ^]^ Copyright 2020, Wiley‐VCH. (B) The highly moisture‐stable performance of TENG sensors based on particle lithography fabrication of 3D layered patterned PDMS. Reproduced with permission.^[^ ^139^ ^]^ Copyright 2019, Elsevier. Finally, (C) presents the preparation process of a self‐powered PENG sensor using electrospinning and spraying technology. These methods highlight the potential for developing high‐performance sensors with diverse functionalities and applications. Reproduced with permission.^[^ ^171^ ^]^ Copyright 2023, Elsevier.

A typical superhydrophobic stretchable strain sensor consists of a conductive polymer composite (CPC) and a constructed superhydrophobic interface.^[^ ^189^ ^]^ The conductive polymer material is typically prepared using dip coating or melt processing by combining a conductive filler and an elastic polymer. Common conductive materials include metal nanoparticles,^[^ ^126^, ^161^, ^163^, ^190^ ^]^ carbon nanomaterials such as carbon nanoparticles, carbon nanotubes,^[^ ^191^, ^192^ ^]^ MXene,^[^ ^115^, ^153^, ^193^ ^]^ and graphene.^[^ ^194^, ^195^ ^]^ These materials serve both as an active sensor network and as the rough topological structure of the superhydrophobic surface. Chen et al. developed a straightforward method to create superhydrophobic strain sensors by swelling and sonicating the surface of rubber bands filled with dense carbon black particles, followed by coating with a layer of low surface energy PDMS.^[^ ^71^ ^]^ Thanks to it, the risks of polymer degradation and filler damage can be avoided, which may severely reduce the stability and durability of sensing devices. Meanwhile, the superhydrophobic wearable sensor in this study can be installed on the human body to detect and quantify motion, even when subjected to the impact of corrosive liquids for more than 8 h. In addition to the long‐term risk of water‐absorbing degradation of polymers or desorption of conductive materials, the operational stability of wearable sensors is also threatened by transient crosstalk caused by liquid attachment. A droplet is shown to be deposited on the surface of a conductive piezoresistive sensor, and this behavior exhibits anti‐crosstalk performance due to the moisture resistance provided by superhydrophobic surfaces. When the surface is untreated, the resting droplet acts as a new liquid resistance, which is connected in parallel with the sensor resistance in the contact part and in series with the sensor resistance that is not in contact with the liquid. This would thus form a new resistance that can affect the sensor's performance and provide fake signals to the electrical terminal. However, for a piezoresistive sensor with a superhydrophobic surface, the micro‐nano structure on the surface creates an air cushion between the solid and liquid, resulting in a small contact area and little impact incurred by the water droplet. This allows the total resistance to remain as the initial resistance. Lin et al. presented a superhydrophobic sensor that exhibits high resistance to liquid crosstalk (Figure 7A).^[^ ^37^ ^]^ The sensor is based on MXene/Graphene/PDMS and features a robust Cassie–Baxter state, which provides exceptional stability to the sensing system. In the majority of studies, the stability of superhydrophobicity has been assessed through techniques such as chemical corrosion and mechanical abrasion. However, given that the formation of hierarchical micro‐/nano‐structures in superhydrophobic surfaces often involves the use of conductive materials, it is important to consider both the stability of superhydrophobicity and the conductivity (sensing performance) when subjected to external aggressions. Dai et al. demonstrated a strong robustness of superhydrophobic surfaces even after immersing in seawater environments for more than 336 h.^[^ ^36^ ^]^ The superhydrophobic and conductive structures were achieved via magnetic field‐assisted self‐assembly strategy, followed with swelling carbon black and superhydrophobic silica nanoparticles for co‐existence of non‐wetting and sensing capabilities.

#### 3.1.2 Self‐powered sensors based on triboelectric nanogenerators

Self‐powered sensors that are based on triboelectric nanogenerators (TENGs) use the universal triboelectric phenomenon, which solves the problem of power supply for distributed sensors. Over the years, both academia and industry have invested considerable effort in improving the performance of TENGs, including energy density, conversion efficiency, working range, and sensitivity when operating as self‐powered sensors.^[^ ^196^ ^]^ However, the stricter environmental application requirements have hindered the practical application of TENG‐based sensing devices. This is primarily due to the formation of a water film on the triboelectric interface, which can significantly degrade the charge transfer or cause charge dissipation under high humidity, as well as the negative impact of dust adhesion on the triboelectric performance. The development of superhydrophobic TENG not only addresses the output degradation of solid‐solid TENG (including unipolar and contact separation) caused by intrusive humidity, but also provides a new approach to generate electricity by utilizing liquid as a friction body in contact with the triboelectric layer. Zhou et al. demonstrated an antifouling superhydrophobic TENG that maintains a stable voltage output even in high humidity environments, which is achieved through the use of a particle lithography‐based 3D layered PDMS film (Figure 7B).^[^ ^139^ ^]^ The reported enhancement of the electrical signal is attributed to the roughness of the superhydrophobic surface, which resulted in a larger contact area and enhanced charge density of the 3D layered structure. The superhydrophobic surface not only ensures the complete removal of liquid from the surface but also enables the bouncing motion of water droplets, making it a key requirement for solid‐solid TENGs. This moisture insensitivity and signal enhancement highlight the importance of superhydrophobicity in the development of TENG‐based devices. Wen et al. demonstrated the feasibility of using superhydrophobic triboelectric nanogenerators on fabric substrates for virtual and augmented reality applications, even in high humidity conditions.^[^ ^197^ ^]^ Besides, the application of fluorination can lead to increased roughness and reduced surface energy, which in turn can provide a larger contact area to enhance the electronegativity and output of triboelectric nanogenerators. Also, Feng et al. developed a sensitive self‐powered sensor for recognizing motion states using a fluorinated polyurethane (F‐PU) layer with surface microcones fabricated through DIL and CVD technologies.^[^ ^198^ ^]^ The enhanced output of the sensor was attributed to the surface modification and the resultant larger contact area. The F‐PU layer served as a superhydrophobic friction point layer and exhibited superior sensitivity when compared with the counterpart that is without the superhydrophobic functions. In summary, superhydrophobic surfaces are of significant importance for wearable devices based on solid–liquid and solid–solid friction nanogenerators. These surfaces offer advantages in both sensing and power supply devices, while also expanding the possibilities of using liquid as a frictional medium. As sensing devices, TENGs with superhydrophobic surfaces exhibit enhanced sensitivity and effectively prevent the intrusion of liquids like sweat or water into the sensing components of wearable devices, thereby minimizing external environmental interference and ensuring accurate and reliable sensing measurements. The self‐cleaning capability further optimizes their lifespan and accuracy, even in the presence of dirt or dust, enabling continuous and reliable sensing performance that is crucial for long‐term monitoring applications. On the other hand, TENGs with superhydrophobic surfaces can also serve as power supply devices for other wearable sensing devices, ensuring efficient energy conversion and reducing the chances of short circuits or performance degradation.

#### 3.1.3 Self‐powered sensors based on piezoelectric nanogenerators

The electric dipole moment in materials contracts when subjected to physical pressure, resulting in the generation of equal positive and negative charges to resist this change. This is the principle behind piezoelectric nanogenerators (PENG) (Figure 7C).^[^ ^171^ ^]^ Nanomaterials with piezoelectric effects are expressed in fiber form, but due to their fragile structure, PENGs have the characteristic of poor mechanical stability and are easily affected by mechanical shocks and exposure to liquids. Traditional PENGs require precise encapsulation, which not only increases processing costs but also reduces the sensitivity of piezoelectric devices due to the excessive thickness of the coating. In contrast, the extreme superhydrophobicity of superhydrophobic PENGs can avoid various chemical corrosion and physical wear. Su et al. developed a superhydrophobic PENG with sweat and moisture resistance by chemically depositing a nano‐coating on the surface of a flexible electrospun piezoelectric nanofiber film.^[^ ^171^ ^]^ As shown in the figure, P(PFDA‐*co*‐EGDA) was co‐deposited coaxially on the surface of the thin film, exhibiting stable voltage signal output not only in high humidity environments but also in corrosive liquids.

### 3.2 Wearable optical sensors

**FIGURE 8:**
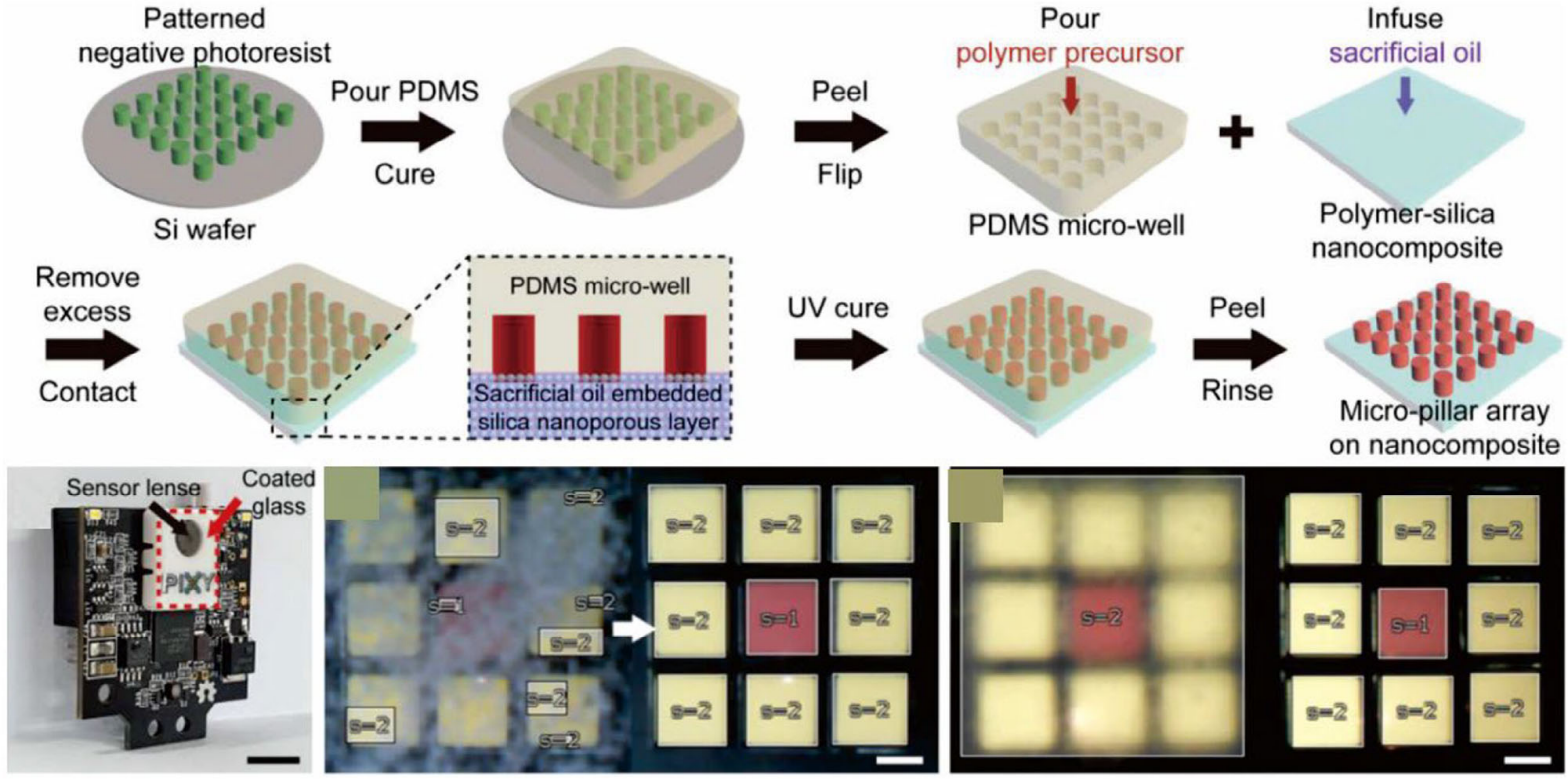
Schematic illustration of a two‐step lithography procedure for transferring a micropillar array onto a polymer‐silica nanocomposite, enabling the fabrication of an antifogging superhydrophobic optical sensor protector. This method highlights the potential for developing advanced protective coatings with enhanced resistance to fogging, and thus improved performance in challenging environments. Reproduced with permission.^[^ ^140^ ^]^ Copyright 2020, Wiley‐VCH.

The field of wearable optical sensing and on‐body display technology is susceptible to the adverse effects of dust adhesion on the surface of wearable devices. This might subsequently result in the decay of luminous flux and transparency, as well as the signal acquisition degradation.^[^ ^136^ ^]^ Consequently, industry and academia are proactively exploring self‐cleaning and antifouling approaches for cases where optically transparency is required. These strategies aim to eliminate fine dust or liquid adsorption by utilizing minute energy inputs from both internal and external sources, preventing device performance degradation. Besides, transparent superhydrophobic surfaces exhibit remarkable anti‐fogging properties.^[^ ^199^ ^]^ Unlike hydrophilic surfaces that can suffer from microscopic droplets condensing and merging into larger droplets under humid conditions that causes changes to light refraction and transparency, microdroplets tend to gather and spontaneously jump off from superhydrophobic surfaces. This attribute is especially valuable for flexible electronics, where the ability to withstand chemical corrosion and mechanical wear is critical. Achieving robust superhydrophobicity while maintaining high transparency is a significant challenge due to the trade‐off between surface roughness and transparency. Wetting theory explains that hydrophobic surfaces have a higher contact angle with increasing surface roughness, but this intensifies light scattering.^[^ ^200^ ^]^ Rayleigh and Mie scattering theories can describe light scattering for different rough surfaces, with Rayleigh scattering being more intense for rougher surfaces with lower particle diameters, while Mie scattering dominates for larger particle diameters. However, Rayleigh scattering has no visible impact when the roughness is less than 100 nm, considering the typical visible light wavelength of 532 nm. The strict constraints on thickness and roughness required for superhydrophobic surfaces pose significant challenges to their stability.^[^ ^201^ ^]^ To address the aforementioned issue, several approaches have been reported for preparation of flexible and transparent superhydrophobic surfaces, including sol‐gel, chemical vapor deposition, template, and phase separation methods. To ensure the stability of superhydrophobic surfaces under various mechanical stresses, spray coating and solution processing are two widely employed techniques. A recent study by Lyu et al. utilized a green water‐bath technique to develop a transparent superhydrophobic coating on epoxy‐coated glass plates based on surface‐treated silica particles.^[^ ^181^ ^]^ The strong interaction between the particles and the resin, combined with the substrate's flexibility, results in a coating with long‐term stability against water impact and chemical corrosion. Lee et al. presented a transparent anti‐fogging superhydrophobic coating that is composed of hydrophobic PFPE micropillars on a silica nano‐porous layer (Figure 8).^[^ ^140^ ^]^ This strategy combines the hydrophilic and superhydrophobic array patterns, utilizing two contrasting properties that exhibit strong anti‐fogging performance to ensure the long‐term transparency and robustness of the optical device surface. The coating displayed remarkable superhydrophobicity even after being subjected to 20 rounds of rubbing with a coarse sandpaper weighted at 50 g, maintaining a stable sliding angle of 10°. The durability of this superhydrophobicity is attributed to the micropillar array support, while the underlying substrate ensures a long‐term transparency.

### 3.3 Wearable chemical sensors

**FIGURE 9:**
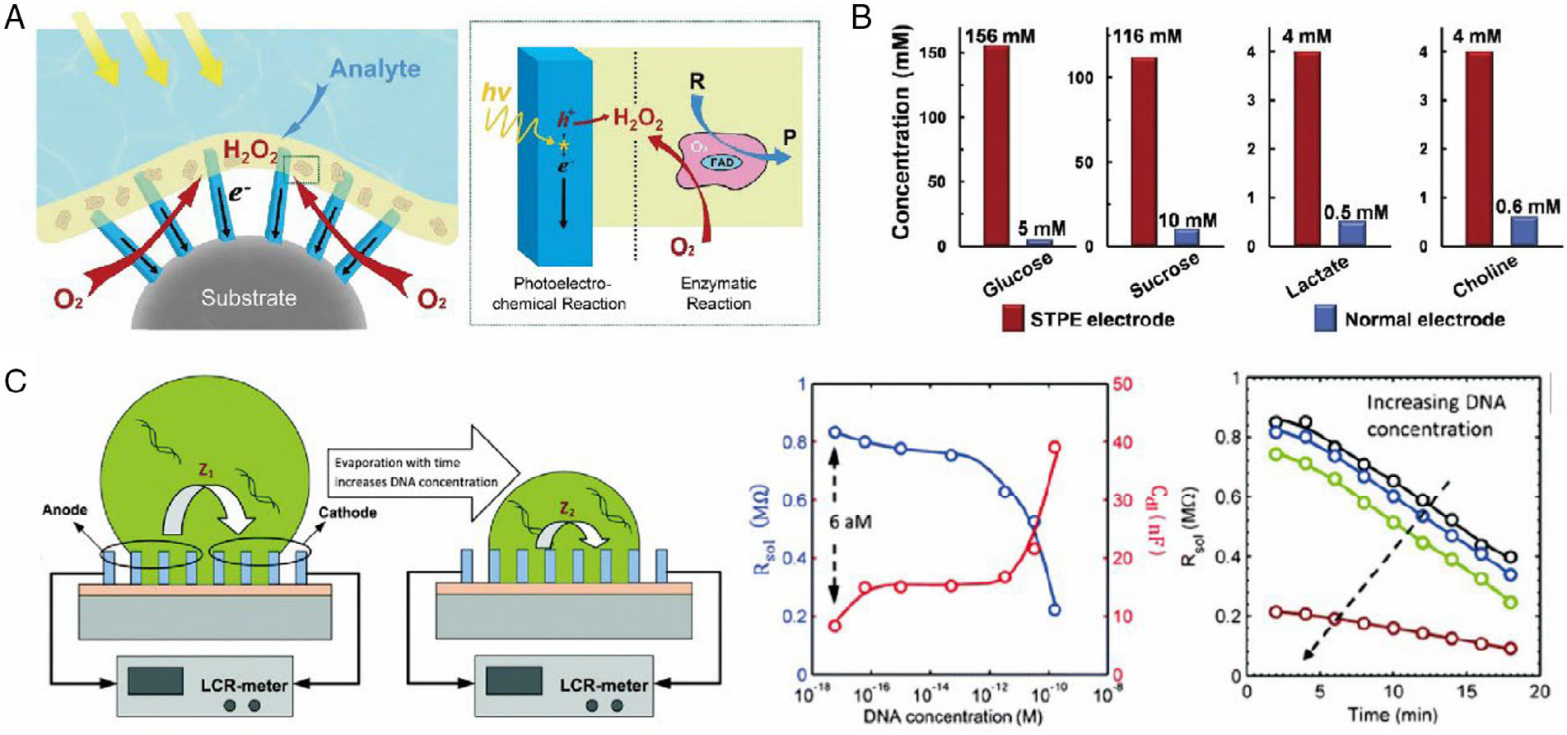
Schematic diagram of the fabrication and performance of advanced superhydrophobic chemical sensors. (A) The schematic diagram of an oxidase/chitosan composite thin layer immobilized on a single crystal TiO_2_ nanowire array film grown on a superhydrophobic substrate. The enlarged view of the reaction zone highlights the benefits brought by air pockets. Reproduced with permission.^[^ ^141^ ^]^ Copyright 2018, Wiley‐VCH. (B) The performance of the developed superhydrophobic chemical sensors in oxidase‐based bioassays, compared with common electrodes. Reproduced with permission.^[^ ^183^ ^]^ Copyright 2016, Wiley‐VCH. (C) Evaporation of a droplet on the nanotextured surface of a nickel electrode leads to an increase in the concentration of guided DNA molecules, reflected in the droplet impedance. Reproduced with permission.^[^ ^142^ ^]^ Copyright 2013, Royal Society of Chemistry.

Chemical sensors are widely used for the qualitative and quantitative detection of various analytes, such as biomarkers, in wearable devices for monitoring vital signs and mobility. These sensors often rely on probes that directly and chemically interact with the target analyte.^[^ ^202^ ^]^ The superhydrophobic surface with a hierarchical micro/nanostructure has been shown to be advantageous for chemical sensors by endowing the probe with gas‐holding ability, which can significantly improve the reaction kinetics of many gas‐based reactions. Chen and colleagues reported on an enzyme biosensor based on a superhydrophobic electrode synthesized by a hydrothermal method (Figure 9A).^[^ ^141^ ^]^ The superhydrophobicity of the electrode allows for rapid gas supply at the solid–liquid–gas three‐phase reaction interface, resulting in high detection efficiency and sensitivity for the analyte glucose. As shown in Figure 9B, Lei and colleagues fabricated electrochemical solid‐contact polymer biosensors by modifying Pt catalysts on conductive superhydrophobic carbon fiber electrode substrates.^[^ ^183^ ^]^ This modification effectively solved the problem of insufficient oxygen that is normally required in the enzymatic reaction zone. In addition, the surface water‐repellent properties result in a substantially reduced droplet contact line. As the concentration of a solution increases, droplets on the superhydrophobic surface in the Cassie–Baxter state will transition to the pinned Wenzel state due to solute precipitation, exhibiting a rapid response that is not limited by diffusion. Ebrahimi et al. conducted research on the detection of DNA concentration at the attomole scale within a droplet (Figure 9C).^[^ ^142^ ^]^ To achieve this goal, they developed a novel method of fabricating superhydrophobic nanotextures by electroplating nickel, which resulted in the immediate localization of target droplets after deposition on the electrode surface. This approach led to a significant improvement in the sensitivity and robustness of the detection process, allowing for the successful detection of DNA at extremely low concentrations. It is because compared to hydrophilic surfaces, the smaller contact line enables solute enrichment in a smaller area, facilitating more precise component detection. Similarly, this enrichment strategy is also achieved through patterned arrays of superhydrophilic and superhydrophobic surfaces. Specifically, small hydrophilic dots are distributed on a superhydrophobic surface to facilitate the localization and concentration of sensing points while mitigating the coffee ring effect. This is also important for applications of wearable chemical sensing, especially for cases with a lower concentration of target analytes.

### 3.4 Wearable electrical sensors

Electrical sensors are mainly applied to detect the changes in skin electrical resistance or changes in skin surface capacitance or conduction coupled charges. Biomedical electrodes, a type of electrical sensor, can convert the ionic potential generated by electrochemical activities into the electronic potential of the measurement system. This measurement system is used to detect various physiological signals such as electrocardiogram, electroencephalogram, electromyography, and nerve potential, which have been extensively applied in clinical medicine and the biological field. Compared to wet electrodes, dry electrodes do not require the use of electrolyte gel, and have the potential for long‐term stable detection. However, the impedance of the skin interface may be influenced by the thickness of sweat and insulating stratum corneum, which can lead to abnormal measurements. To address this issue, dry electrode arrays based on superhydrophobic materials can maintain good electrode–skin contact conditions for a longer duration. This can lead to an improvement in the stability of the measurement process when using dry bioelectrodes. As shown in Figure 6E, Zhou and colleagues conducted a study on modifying the surface of copper electrodes with complex textures using laser processing and dip coating techniques, with the aim of obtaining superamphiphobic properties through stearic acid modification.^[^ ^143^ ^]^ The modified surface exhibited repellency towards glycerin, water, and sweat, indicating the successful achievement of superamphiphobicity that prompts the stable signal collections.

## 4 EVALUATION OF ROBUSTNESS

Despite the significant contribution of the concept of superhydrophobicity in enhancing the stability and functionality of flexible wearable electronics for operations in complex environments, the practical implementation in the industry has not been entirely satisfactory.^[^ ^208^ ^]^ Although various synthesis and fabrication techniques have been developed to achieve superhydrophobicity, the performance degradation in real‐world applications remains a major challenge. This is primarily due to the unavoidable wear, corrosion, and aging of superhydrophobic surfaces that are based on low surface energy materials and hierarchical micro/nano‐textures. Furthermore, the repeated deformation of flexible substrates might bring the concerns of preserved non‐wetting capability, leading to unpredictable degradation of the performance of flexible wearable devices. In addition to the irreversible performance decay as mentioned above, the superhydrophobic function of wearable electronics might also be affected via other external stimuli, for example, the mechanical deformation. During this deformation process, the expanded area of the flexible substrate may cause changes in the spacing of surface features, leading to wettability transitions and resulting in sensing failure. The mechanical instability is primarily due to the highly layered texture structure of superhydrophobic surfaces. This characteristic leads to concentrated load in the contact area, resulting in the structural collapse due to the applied mechanical stimuli. The fragility is unacceptable for applications in wearable sensing or interactive human‐machine interfaces. Moreover, superhydrophobicity can be permanently lost when subjected to high levels of chemical abrasion. Additionally, under conditions of high humidity and water pressure, the transformation from the Cassie state to the Wenzel state is an interesting phenomenon to explore and understand the mechanisms of energy and force‐based destruction. This section aims to provide a comprehensive summary of common methods of testing and characterizing the superhydrophobic robustness and potential failure mechanisms in specific environments. In Table 2, based on the practical durability testing objectives for different types of superhydrophobic sensors, we have categorized and provided commonly used methods for testing the robustness of superhydrophobic surfaces. Additionally, the testing strategies for the development of superhydrophobic wearable sensors with long‐term stability and environmental adaptability are also presented in the following sections. These testing methods must assess not only the robustness of superhydrophobicity, but also the stable signal output from the sensing components due to the coupling design.

**TABLE 2:** Summary of evaluation methods on durable superhydrophobic surfaces.

Methods	Key parameters	Purpose	Target surface	Application‐oriented testing	Ref.
Tape peeling: A tape is applied on the surface of the tested material, and it is pressed (or even loaded with objects). Then the tape is peeled from one end	Adhesion force of the tape to a reference substrate, the weight of the load, distance	Adhesive durability	Superhydrophobic coating	Surfaces involved in contact and friction	[203, 209]
Cross‐cut test: Scribes the target area of the film, forming a square grid or X shape (can be used before the peel‐tape test)	Standard ASTM D3359	Brittleness adhesive durability	Superhydrophobic coating, flexible superhydrophobic film	Surfaces involved in contact and friction that are often subjected to tangential forces	[210, 211]
Ultrasonic treatment: Ultrasonic treatment of superhydrophobic surfaces in liquid at a certain frequency	Frequency, ultrasonication time, liquid medium	Mechanical durability	Generally applicable	Common surfaces that need to be washed and cleaned	[116, 212]
Abrasion: a horizontal arm holding a vertical cylinder covered with different abradant that reciprocates or perform a rotational motion in a linear direction with certain speed and the length	Abradant, speed/angular velocity, length (cycle), pressure to the superhydrophobic surface	Tangential abrasion durability	Generally applicable	Surfaces involved in contact and friction	[39, 213]
Knife test: Linear abrasion of the surface with a sharp blade	Length, pressure to the superhydrophobic surface	Tangential abrasion durability	Generally applicable	Surfaces involved in contact and friction	[214, 215]
Oscillating steel test: A steel ball or ring is brought into contact with the sample surface and pressurized with a gradually increasing normal load until the surface features are destroyed	Pressure to the superhydrophobic surface, oscillating frequency	Mechanical durability	Superhydrophobic surface with micro‐nano array structure	Surfaces involved in contact and friction	[215, 216]
Pencil test: Drag a pencil with quantified hardness on the surface	Pencil hardness, speed, and pressure to the superhydrophobic surface	Tangential abrasion durability	Generally applicable	Surfaces in contact with hard surfaces	[217, 218]
Rotary slurry test: Spin a propeller with a superhydrophobic surface in a slurry	Composition, size and shape of particles in the slurry, rotational speed	Mechanical durability	Generally applicable	Common surfaces that need to be washed and cleaned	[219, 220]
Solid particle impact: Continuous vertical impact and contamination on inclined superhydrophobic surfaces with gravel	Sand particle density/radius, exposure time	Recommendations for tangential abrasion	Outdoor application	Surfaces for Outdoor Environments	[221, 222]
Liquid jet/droplet impact: Eject droplets or water jets on superhydrophobic surfaces	Droplet size, impact velocity, exposure time	Recommendations for tangential abrasion	Outdoor application	Surfaces for outdoor environments	[223, 224]
Finger press: Press or reciprocate a bare finger against a surface with some force	Length, speed, pressure to the superhydrophobic surface	Mechanical durability	Superhydrophobic surfaces involved in industrial applications	Surfaces that often come into contact with human body surfaces	[225, 226]
Washing test: The laundry test	Type and amount of detergent, washing time, addition of abrasives	Mechanical durability	Superhydrophobic fiber or flexible film	Common surfaces that need to be washed and cleaned	[227, 228]
Chemical corrosion: Immersion of superhydrophobic surfaces into corrosive liquid environments containing acids/alkali/salts	Duration, solution concentration	Chemical corrosion durability	Generally applicable	Protective film, outdoor and other extreme scenes	[229, 230]
UV radiation: Simulation of UV light exposure to surfaces by environmental testing or aging chambers	Duration, irradiation intensity	Environment durability	Generally applicable	Surfaces for outdoor environments	[205, 231]

### 4.1 Mechanical wear resistance

High‐density mechanical loads acting on the textured surface inevitably pose a threat to the superhydrophobic performance. Taking advantage of this common defect, mechanical wear tests include a variety of strategies to evaluate the robustness of superhydrophobic surface. In principle, the robust superhydrophobic strategy is designed to enhance the adhesion between the functional coating and the supporting substrate layer. Furthermore, the capability to actively regenerate or passively repair the microstructure and low surface energy materials is another aspect to evaluate the potential for long‐term applications. Mechanical stability testing protocols are commonly used to evaluate these two points, and their applicability to different flexible devices is summarized in this section.

#### 4.1.1 Adhesion test

**FIGURE 10:**
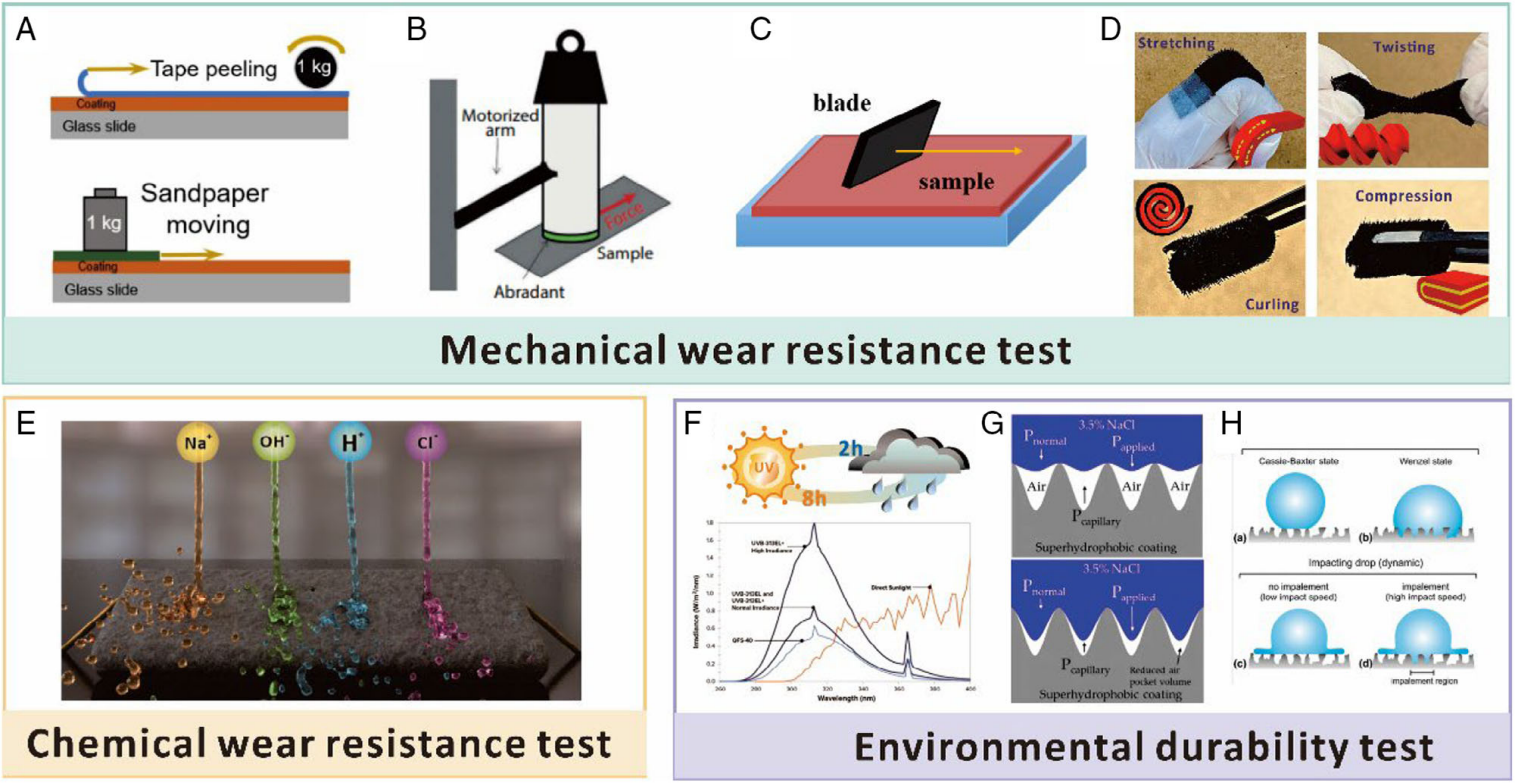
Common practical durability tests for superhydrophobic surfaces typically include mechanical abrasion durability, chemical corrosion durability, and environmental durability. These tests are crucial for assessing the long‐term stability and performance of superhydrophobic surfaces in real‐world conditions. Linear wear test: (A) Tape peeling test. Reproduced with permission.^[^ ^203^ ^]^ Copyright 2020, Elsevier. (B) Standardized mechanical wear, Reproduced with permission.^[^ ^204^ ^]^ Copyright 2015, Wiley‐VCH. (C) Knife edge test, (D) flexibility test, Reproduced with permission.^[^ ^47^ ^]^ Copyright 2022, Elsevier. (E) Chemical test including acid, alkali, salt, Reproduced with permission.^[^ ^205^ ^]^ Copyright 2022. (F) UV aging test. Reproduced with permission.^[^ ^205^ ^]^ Copyright 2022, Elsevier. (G) Hydrostatic pressure test. Reproduced with permission.^[^ ^206^ ^]^ Copyright 2018, American Chemical Society. (H) Dynamic test. Reproduced with permission.^[^ ^207^ ^]^ Copyright 2022, Elsevier.

The objective of the adhesion test is to evaluate the bonding strength between the superhydrophobic coating and the surface of flexible electronics, which is often formed by common techniques such as dip coating, spray coating, scrape coating, and spin coating. Since the desorption of the coating may expose the non‐wetting structure, strategies such as cross‐linking and covalent bonding are often utilized to improve the adhesion between the coating and the substrate for a higher robustness. As a result, adhesion testing is commonly performed by applying peeling forces and measuring the energy required to detach the surface coating. One widely used method for testing superhydrophobic surfaces is Tape peeling (Figure 10A). This involves applying a tape with standard adhesion to the surface of the sample to be tested, and then applying a specific pressure, either statically or dynamically, to ensure that the tape is in close contact with the coating surface. The tape is then peeled from the substrate. After conducting these tests, the superhydrophobic properties of the surfaces can be evaluated using contact angle and rolling angle tests. In this test scheme, the parameters used to evaluate the stability of superhydrophobic mainly include adhesion force of the tape to a reference substrate, as well as the weight of the load and the number of cycles of adhesion. The tape peeling method is a direct measure of the bonding strength between the superhydrophobic surface coating and its microstructure. Cao et al. developed a superhydrophobic coating with high substrate adhesion by incorporating supramolecular organosilicon polymers and silica nanoparticles, which allowed for a rapid damage repair mechanism based on dynamic chemical bonds.^[^ ^203^ ^]^ The strength of the binding force was evaluated using the tape peeling test with a load exceeding 1000 g for 40 cycles. To conduct a more rigorous test, the standard ASTM D3359 is often referenced, and a cross‐cut treatment is performed prior to the tape peeling test. Cross‐cutting involves cutting the target area of the film to form a square grid or X shape, which aids in the detachment of the coating and facilitates the degradation caused by subsequent tape stripping. Furthermore, when assessing wearable sensors attached with nano‐active materials, such as piezoresistive sensors and chemical sensors, it is important to consider that the tape peeling test may cause desorption of the active materials and result in performance degradation. Therefore, it is necessary to conduct relevant property checks simultaneously to accurately evaluate the performance of the sensors. On the other hand, ultrasonic testing has the potential to induce the desorption of surface energy substances or nanostructures in wettable solutions by external energy.^[^ ^232^ ^]^ Ethanol, unlike water with a much higher surface tension of 72.8 mN m^−1^, is capable of wetting most superhydrophobic surfaces because of a smaller surface tension of 22.55 mN m^−1^. Consequently, ethanol is often used as a liquid medium for ultrasonic testing to assess the robustness of superhydrophobic surfaces. In addition to the medium, ultrasound power and time are also important evaluation parameters. Yin et al. utilized the ultrasonic method to assess the stability of a released superhydrophobic surface, using ethanol and n‐hexane as liquid media.^[^ ^233^ ^]^ Their results indicate that the 509 reagent@Cu nanoparticles/Cu mesh/Cu organic‐inorganic multilayer superhydrophobic coating exhibited strong robustness, with a contact angle decrease of less than 10° during the first 50 min of ultrasonic treatment.

#### 4.1.2 Hardness of structure

Robustness testing is crucial for wearable electronic devices with superhydrophobic surfaces because they are generally exposed to various external stimuli during the applications such as human–computer interaction devices. The stability of superhydrophobic surfaces is affected by tangential abrasion and normal pressure, which cause changes in surface morphology and chemistry due to mechanical wear, often assessed based on accumulated loads. Various methods have been employed for evaluating tangential wear. One common method is by rubbing sandpaper loaded with heavy objects on the superhydrophobic surface to intuitively reflect its firmness. In this model, the grit of the sandpaper, the size of the load, and the wear cycle are important parameters. Several reviews have pointed out the subjective nature of force application during sandpaper abrasion, leading to difficulties in accurately quantifying the degree of robustness of superhydrophobic surfaces. To overcome this limitation, a tangential wear evaluation instrument with wide applicability has been developed. The standard abrasion configurations of this instrument involve a motorized arm that moves and rubs the tested sample surface tangentially or circumferentially. The evaluation parameters include abradant, speed/angular velocity, length (cycle), and the size of the load. Using this instrument, Wang et al. evaluated the robustness of their superhydrophobic surface prepared by the armor strategy.^[^ ^39^ ^]^ The surface maintained a static contact angle greater than 150° even after more than 1000 reciprocating linear abrasions at 12 MPa, demonstrating remarkable durability (Figure 10B). Scratching the superhydrophobic surface with a sharp implement, such as a knife as shown in Figure 10C, is a widely used method for tangential wear assessment. This method is specifically designed to target the micro‐nanostructures of the surface and can effectively evaluate both the adhesion performance and the structural stiffness. The key evaluation parameters of this method include the angle of the blade, the load, and the speed of the scratch. Furthermore, to evaluate superhydrophobic surfaces with micro‐nano array characteristics, the oscillating steel test is commonly employed.^[^ ^214^, ^215^ ^]^ Hensel et al. used this method to assess the mechanical durability of their hydrophobic polymer membranes.^[^ ^234^ ^]^ This test involves bringing an oscillating steel ball into contact with the sample surface and gradually increasing the normal load until the surface characteristic is destroyed. Besides, pencil hardness is another commonly used method to evaluate the durability of superhydrophobic surfaces. Due to their marked hardness, pencils are rolled over the surface to be measured with a constant contact force. The microstructure is then observed through electron microscopy to determine the integrity of the superhydrophobic surface. For instance, Chen et al. reported a superhydrophobic coating based on methyl silicone resin and superhydrophobic silica sol that passed the 9H pencil hardness test, which is much larger than the common 5H.^[^ ^235^ ^]^ This indicates the rigidity of the structure and its ability to withstand external forces. The propeller test can also be used which involves the collision of particles in the slurry with the superhydrophobic surface during propeller rotation. This method is useful for testing the durability of superhydrophobic coatings under conditions similar to those encountered in industrial applications. The propeller rotation speed, the concentration and size of particles in the slurry, and the duration of the test are the main parameters evaluated in this method.^[^ ^219^ ^]^ Considering the practicability of superhydrophobic flexible and wearable sensors, the above methods can serve as important standards to evaluate the robustness of flexible superhydrophobic interface for wearable electronics.

#### 4.1.3 Application‐oriented testing

In the evaluation of superhydrophobic surfaces, application‐oriented tests are commonly conducted to assess the performance of superhydrophobic surfaces in specific scenarios. To evaluate the performance of superhydrophobic surfaces under outdoor conditions, it is essential to consider the dynamic effects that the surface may encounter. These effects can include wind, rain, and other environmental factors that can affect the stability of the surface.^[^ ^236^ ^]^ For example, wind can cause particles to impact the surface, which can lead to wear and tear over time. Besides, the impact of raindrops with potential energy can cause a transition from the Cassie state to the Wenzel state by impacting the liquid/air interface. The impact testing of sand particles is often necessary as the effect of impact on the stability of the structure as well as the self‐cleaning properties can be evaluated simultaneously. Similarly, water‐based dynamic tests, such as water impact and droplet rebound tests, can simulate the non‐wetting state of superhydrophobic surfaces under conditions similar to rain or other dynamic situations.^[^ ^237^ ^]^ As a means of simulating human interaction with superhydrophobic surfaces, finger press test is also one common method that has been widely introduced.^[^ ^238^ ^]^ However, the adhesion of materials, for example, salt and oil pollutants, may cause performance degradation as well as the collapse of surface structures. Therefore, this test can evaluate the robustness of superhydrophobic surfaces and provide performance criterion for a touchable device. In addition, for extensive fabric‐based flexible electronics, the washing method is also an important evaluation technique to meet common practical needs in daily life.^[^ ^227^ ^]^ The addition of washing liquid can simulate the actual washing process, and the introduction of water temperature and steel balls can further accelerate the wear process. This method can assess the durability and stability of the superhydrophobic coating under repeated washing cycles and provide insights into the development of more robust and reliable coatings for superhydrophobic flexible electronics.

Given that wearable sensors are invariably affixed to human skin, they frequently undergo mechanical deformations such as bending, twisting, and stretching. In the context of superhydrophobicity, two key variables, surface morphologies and low‐surface‐energy properties, are susceptible to alterations under these mechanical perturbations. Such alterations raise significant concerns regarding potential decay in superhydrophobicity and unanticipated wetting transitions.^[^ ^97^, ^239^ ^]^ Therefore, the robustness evaluations of superhydrophobic flexible electronic surfaces are also required under various flexible tests such as tensile, torsion, and bending (Figure 10D).^[^ ^47^ ^]^ Liu et al. achieved wearable superhydrophobic strain sensors by depositing CNC/G coatings on non‐woven fabrics and introducing hydrophobic nano‐silica dioxide. Due to the accumulation of micro‐nanostructures, droplets on the sensor's surface still maintain a cap‐like shape even under 80% elongation. The deformation insensitivity of such superhydrophobic surfaces ensures excellent water repellency and anti‐interference throughout the sensing process, even when subjected to liquid environments.^[^ ^240^ ^]^ Such assessments are pivotal in gauging the resilience of flexible superhydrophobic interfaces under physical stresses. In the tensile test, the specimen undergoes tensile forces that can evaluate the endurance against tension and the superhydrophobic preservation. Conversely, torsion tests involve subjecting the sample to twisting forces, ascertaining its resistance to rotational perturbations. Bending tests, on the other hand, assess the efficacy of the superhydrophobic surface when contorted. To summarize, these evaluations ascertain the integrity of surface superhydrophobicity under diverse mechanical stresses, thereby furnishing data on long‐term stability and functionality. The execution of such tests typically involves using motors to simulate the deformations in real‐world applications. The degree of wear and aging is calibrated based on the number of cycles, and subsequently, general tests are conducted on the sensors to assess their performance and superhydrophobic properties. Beyond post‐deformation evaluations, dynamic assessments during the deformation phase are equally critical. Wearable electronics, especially those adhering to the skin's contours, are frequently utilized to monitor joint movements, which inevitably exposes them to deformations. In such scenarios, singular‐scale roughness may facilitate the spread of liquids across the device's surface and the non‐wetting property would be lost. This can finally result in potential corrosion, short‐circuiting, or other interferences. Therefore, the potential degradation caused by these two types of deformations must be considered, and relevant tests are extremely important before the practical application of flexible electronic products.

### 4.2 Resistance of chemical corrosion

While superhydrophobicity can utilize the air cavities in the three‐phase interface to minimize the contact area between corrosive liquids, ultimately long‐term exposure may still cause the decay of superhydrophobicity. In the case of flexible electronics, the risk of internal (skin side) corrosion is mainly due to the presence of salt solution (Figure 10E). In addition, surface microorganisms and their metabolites can also cause contamination. On the other hand, for external risks, flexible electronics with superhydrophobic surfaces have potential applications in corrosive liquids. As such, to evaluate the long‐term stability, chemical corrosion resistance testing is commonly used to evaluate the stability of superhydrophobic surfaces that have been exposed to corrosive liquids, including acidic, alkaline, and salty solutions.^[^ ^241^ ^]^ A typical approach is to soak superhydrophobic materials in solutions with a wide range of pH values for a designated period of time and subsequently measure the wettability to determine the behavior of superhydrophobic resistance. Saddiqi et al. used a droplet‐assisted growth method to prepare superhydrophobic surfaces based on organosilicon nanowires, resulting in significantly improved chemical corrosion resistance.^[^ ^242^ ^]^ In a 16‐h underwater test, the proposed coating was kept stable for both superhydrophobicity and conductivity. In addition, the coating was subjected to acidic (pH = 2) and basic (pH = 14) solutions for seven days, and the three‐phase interface was maintained as evidenced by measurements of the apparent contact angle and rolling angle.

### 4.3 Environmental durability tests

The environmental test aims to evaluate the potential threat posed by the external environment to the superhydrophobic surface in various scenarios. One important aspect of this evaluation is the ultraviolet test, which assesses the robustness of the superhydrophobic surface in terms of photoaging. This test involves exposing the superhydrophobic material to ultraviolet light for a certain period and studying the changes in wettability before and after the photoaging process. For instance, Ren et al. prepared a superhydrophobic fabric and subjected it to continuous irradiation for 75 h under a laboratory‐scaled ultraviolet lamp, and demonstrated its stability in terms of contact angle and rolling angle variations (Figure 10F).^[^ ^131^ ^]^ Additionally, outdoor experiments on superhydrophobic fabrics were applied by exposing the samples to direct sunlight for 30 days. In the case of wearable sensors, the flexible substrates, for example, rubber, are susceptible to becoming brittle and losing their flexibility during photoaging. Therefore, it is also important to evaluate their mechanical properties in addition to their superhydrophobicity. Superhydrophobic surfaces can experience failure in high water pressure conditions, as the air layer trapped by the textured surface may collapse due to increased internal hydrostatic pressure caused by external forces.^[^ ^50^ ^]^ The transition from the Cassie state to the Wenzel state can also occur if droplet size becomes smaller than the gaps among micro‐structures, posing a risk in high‐humidity environments (Figure 10G,H).^[^ ^243^ ^]^ Interestingly, certain devices can mitigate decay in high‐humidity environments due to the intrinsic properties of their materials. Liu and colleagues reported a superhydrophobic sensor based on an MXene‐sodium alginate sponge. When a voltage is applied for sensing, the Joule heating effect occurs to reduce the moisture adsorption. This creates a persistent superhydrophobic barrier to ensure stable sensing in highly humid conditions.^[^ ^115^ ^]^ In cold environments, the superhydrophobicity of surfaces reduces the contact area and duration of water, thus reducing the chances of ice formation. However, when droplets remain on such surfaces, condensation can still occur in a delayed freezing time. During this process, water droplets condense on the superhydrophobic surface and penetrate the rough structure, resulting in a transition to the Wenzel state. Consequently, the contact angle hysteresis increases and the superhydrophobicity will lose obviously. Additionally, when the condensed water within the surface structure freezes, the resulting ice mechanically adheres to the surface, creating greater ice adhesion.^[^ ^244^ ^]^ The aforementioned evaluations are crucial for analyzing the potential operating range of flexible wearable devices with superhydrophobic surfaces. By identifying the limitations of these surfaces under different environmental conditions, such as high humidity, UV exposure, and cold environments, researchers can improve the durability and reliability of these devices. In addition, ongoing optimization efforts, such as reentrant structures, can further enhance the performance of superhydrophobic surfaces with advanced capability such as oleophobicity. In addition to targeted testing, the long‐term monitoring performance of human activities in practical application scenarios also needs to be considered to meet the requirements of actual usage. For instance, detection of human body motions often requires continuous monitoring over extended periods, particularly in the context of chronic disease management, health status tracking, and exercise controls. Therefore, the performance of superhydrophobic sensors in terms of long‐term functionality directly impacts their reliability and usability in practical applications. Such testing can identify potential issues through data accuracy, human adaptability, stability, and durability analyses. This is crucial for achieving reliable human monitoring and enhancing the prospects of superhydrophobic sensors in practical applications with further improved performances. By conducting these evaluations and continuously optimizing the design of superhydrophobic surfaces, researchers can advance the development of flexible wearable devices that are more resilient and suitable for a wider range of applications.

## 5 CONCLUSION AND OUTLOOKS

The last decade has witnessed great breakthroughs in the development of flexible and wearable sensors. Especially, the coupling of flexible and wearable devices with superhydrophobic functions exhibits the potential towards a wider range of applications. The fusion of flexibility and superhydrophobicity offers superiorities including safeguarding the internal electronics from moisture and other environmental pollutants. Such behavior is especially crucial for wearable sensors, which are often utilized in outdoor or athletic environments that could expose them to liquids such as rain, sweat, or other similar conditions. Furthermore, the superhydrophobic surface can enhance the robustness and durability of wearable sensors. By reducing the contact area between the sensor and its surrounding environment, the superhydrophobic surface minimizes wear and tear, thereby reducing the risk of damage due to moisture, dirt, or other environmental factors. Another advantage of superhydrophobic surfaces is their potential to enhance the accuracy and reliability of wearable sensors. By decreasing the contact area between the sensor and the skin or other surface, the superhydrophobic surface can minimize errors or interference caused by changes in the surrounding environment, such as variations in temperature, humidity, or surrounding air flow. In addition, superhydrophobic surfaces can increase the comfort and convenience of wearable sensors for users. By minimizing the contact area between the sensor and the skin, the superhydrophobic surface minimizes irritation or discomfort, making the sensor more comfortable to wear for extended periods of time. In summary, the adoption of superhydrophobic surfaces in flexible electronics can lead to several advantages in wearable sensor applications, such as protection against environmental pollutants, enhanced durability, improved accuracy and reliability, and increased user comfort and convenience. It can be expected that the superhydrophobic surfaces could play a significant role in advancing the field of wearable sensors, and further research and development in this area are highly encouraged. After reviewing current literature, we have identified several mainstream applications of long‐term stable flexible electronics with superhydrophobic surfaces in the field of wearable sensors. These potential applications include:

(1) Health monitoring: Superhydrophobic sensors can accurately and reliably monitor various health indicators, such as heart rate, blood pressure, and body temperature, to improve the diagnosis and treatment of numerous health conditions.
(2) Motion tracking: Superhydrophobic sensors can track physical activity and fitness levels, providing users with real‐time feedback on their performance and progress. This can motivate users to maintain an active lifestyle and improve their overall health and well‐being.
(3) Environmental monitoring: Superhydrophobic sensors can monitor environmental conditions, such as air quality, temperature, and humidity, in industrial or hazardous environments where traditional sensors may be at risk of damage from moisture or other contaminants.
(4) Prosthetics and assistive devices: Superhydrophobic wearable sensors with broad response mechanisms can be integrated into prosthetic limbs and other assistive devices to improve accuracy, reliability, and functionality, thereby improving the quality of life for people with disabilities.
(5) Special rescue: Superhydrophobic sensors can be integrated into special rescue equipment such as diving suits, life jackets, and rafts to improve their performance and safety by repelling water and other liquids, preventing equipment from becoming waterlogged. The coupling of wearable devices with superhydrophobicity can potentially reduce the risk of accidents and improve the chances of survival in emergency situations.
(6) Human–machine interaction system: Superhydrophobic sensors can be used to create advanced human‐machine interfaces, such as touchscreens and wearable input devices. By providing a reliable and durable surface that can withstand moisture and other contaminants, superhydrophobic surfaces can improve the performance and longevity of these devices for a more practical and user‐friendly experience. In addition to the sensor component itself, further advancements in superhydrophobic surfaces should also consider the integration of complementary modules such as signal transmission, data processing, and power supply, etc. These combinational systems can offer potential advantages in human–machine applications for future wearable technology with seamless integration and performance optimization across multiple modules.

To conclude, the combination of flexible electronics with superhydrophobic functions offer several potential superiorities for wearable sensors. However, their development and implementation also face challenges and limitations that need to be addressed before commercial consideration. One significant challenge is the development of materials and manufacturing processes that can produce superhydrophobic surfaces with the required properties reliably. Although significant progress has been made in this area, the dynamic mechanisms of wetting transitions in various environments make it challenging to practically apply superhydrophobic surfaces to flexible electronics. Another challenge is the need for rigorous testing and evaluation of these technologies under a broad range of environmental conditions. This includes testing them under exposure to moisture, temperature changes, and other factors that may affect their durability and performance. Standardized testing protocols and validation methods must be developed to provide reliable and accurate data on the performance of these devices in real‐world scenarios. Finally, there is also a need for continuous innovation and development of new applications and use cases for flexible electronics with superhydrophobic surfaces. We also noticed that some wearable devices could function well even under the humid conditions based on the intrinsic working mechanism, for example, the electromagnetic induction, or using the encapsulation technology. This behavior can somehow relieve the fabrication methodology, however, the intrinsic capability normally cannot ensure the extreme resistance for a broader applicable spectrum such as corrosive cases. Superhydrophobic surfaces can greatly facilitate the modular design of wearable devices, benefiting from encapsulation‐free technology to make the overall system more compact.^[^ ^245^, ^246^, ^247^ ^]^ Furthermore, this strategy would enable the multimodal sensing that can be possibly applied for various environments for a more intelligent future.

**FIGURE 11:**
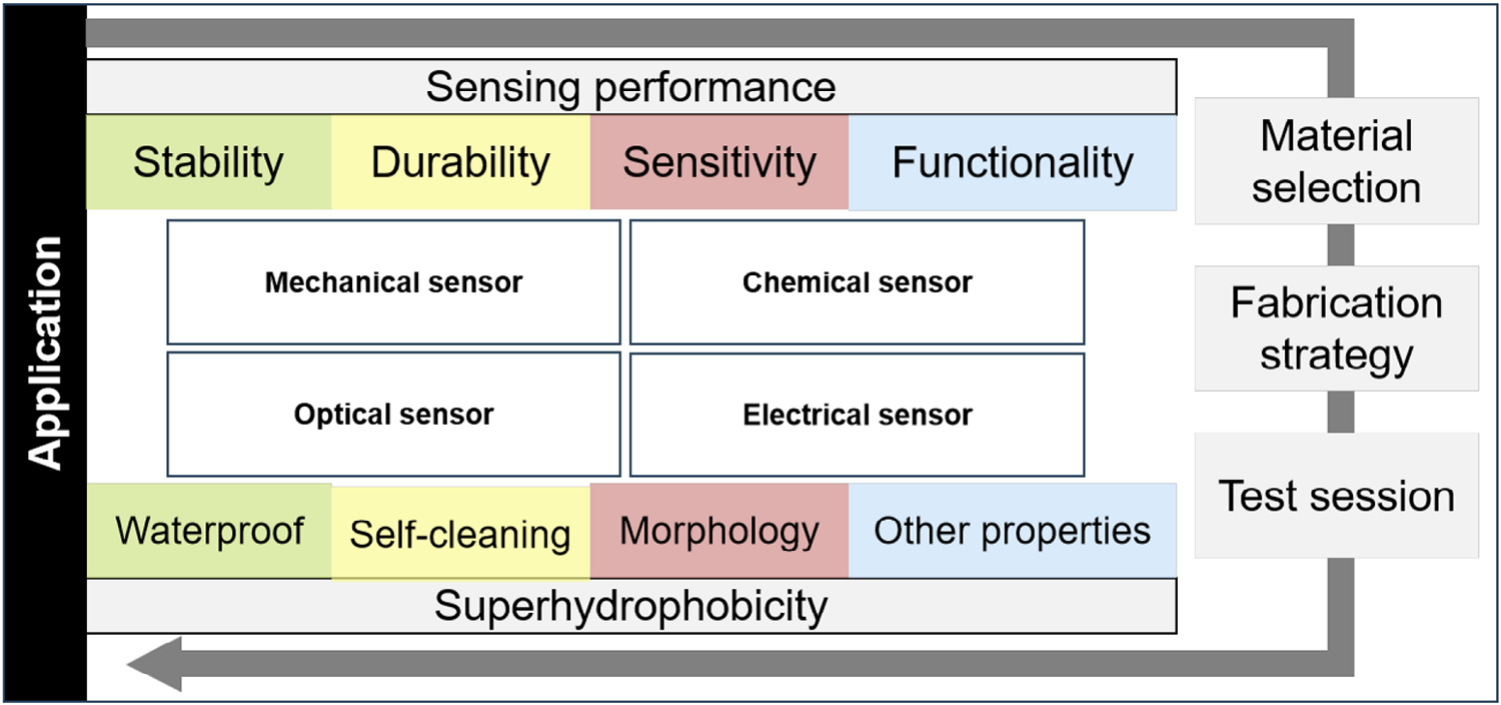
Schematic diagram of the design methodologies for application‐oriented superhydrophobic and flexible sensors.

In other words, wearable electronic devices with superhydrophobic surfaces are facing integrated challenges that necessitate a comprehensive discussion from a systemic perspective. The aspects of material selection, fabrication methods, performance, and applications‐oriented design are required to consider simultaneously to achieve a superhydrophobic wearable sensor for industrial use. Figure 11 elucidates a comprehensive diagram of the design process tailored for application‐centric superhydrophobic sensor devices. This schema spans from the materials selection to fabrication, and robustness evaluation. Given the critical roles of superhydrophobic interfaces in flexible devices, the diagram reveals the tight relationship between the superhydrophobic behavior and the sensing performance. No matter the type of the wearable devices, coupling between non‐wetting behavior and sensor performance should be continuously optimized throughout the fabrication and characterization processes.

With the recent development of superhydrophobic wearable sensors, it is now evident that wearable devices with superhydrophobic functions are of significant potential for diverse applications. These range from pervasive human‐machine interfaces to advanced sensing frameworks and communication methods, which further solidifies the advancements in cutting‐edge virtual reality technologies. Addressing the challenges in this field requires a comprehensive and interdisciplinary approach based on materials science, surface engineering, manufacturing paradigms, device structures, and application scenario‐oriented viewpoints. Overcoming these barriers will pave the way for the emergence of robust and efficient wearable electronics endowed with superhydrophobic attributes. Consequently, this visionary trajectory demands intensive collaborations among academics, industry professionals, and consumers from various sectors such as chemistry, physics, chemical engineering, and biosciences, thus fostering a practical strategy to explore new opportunities and resolve emergent challenges for wearable sensors with superhydrophobic surfaces.

## CONFLICT OF INTEREST STATEMENT

The authors declare no conflicts of interest.
